# The in vitro alkaline comet assay with liver models as a complementary tool for genotoxicity assessment of *N*-nitrosamines

**DOI:** 10.1007/s00204-026-04457-1

**Published:** 2026-06-02

**Authors:** M. A. Djuari, A. Londenberg, A. Bassan, K. Cross, L. Elenschneider, S. E. Escher, J. Fahrer, J. Fangmann, C. Felske, R. Frötschl, B. Haas, G. E. Johnson, M. Vogel, R. Whomsley, C. Ziemann

**Affiliations:** 1 Fraunhofer Institute for Toxicology and Experimental Medicine ITEM, Hannover, Germany; 2 Innovatune Srl, Padua, Italy; 3 INSTEM, Columbus, OH USA; 4 Division of Food Chemistry and Toxicology, Department of Chemistry, Rheinland-Pfälzische Technische Universität (RPTU) Kaiserslautern-Landau, Kaiserslautern, Germany; 5 LiverCenter Hannover (LCH), KRH Klinikum Region Hannover, Hannover, Germany; 6 Federal Institute for Drugs and Medical Devices (BfArM), Bonn, Germany; 7 Swansea University Medical School, Wales, UK; 8 European Medicines Agency, Amsterdam, Netherlands

**Keywords:** Alkaline comet assay, In vitro, Liver, *N*-nitrosamines, Benchmark dose modeling

## Abstract

*N*-nitrosamine (NA) impurities in pharmaceuticals represent “cohort of concern” compounds under ICH M7(R2), due to their mutagenic/carcinogenic potential, involving cytochrome P450 (CYP)-mediated metabolic activation. Increasing interest in mammalian cell-based genotoxicity/mutagenicity assays prompted our assessment of the in vitro alkaline comet assay regarding its predictive power for NAs. Here, precision-cut liver slices (PCLiS), primary human hepatocytes (PHH), primary rat hepatocytes (PRH), and HepG2 cells with rat or hamster S9-mix were investigated as in vitro model systems. Metabolic competence was characterized beforehand. For performance evaluation, a panel of known-mutagenic [*N*-nitroso-dimethylamine (NDMA), *N*-nitroso-diethanolamine, *N*-nitroso-methylaniline, *S*–*N*-nitroso-nornicotine, N-methyl-N-nitroso-2-propanamine] and reported non-mutagenic (methyl-t-butylnitrosamine, *N*-nitrosoproline) was tested, together with Nitrosamine Drug Substance-Related Impurities [*N*-nitrosodesloratadine, *N*-nitrosofolic acid, *N*-nitrosofluoxetine (NFluo)] at a concentration range of 0.005–10 mM. After 2 h (PCLiS, PHH and PRH) or 4 h (HepG2), NDMA concentration-dependently induced DNA strand breaks in all in vitro models. Sensitivity/specificity of the various liver cell models for prediction of carcinogenic NAs were 100%/50% (HepG2 with hamster S9-mix), 50%/100% (PHH, PRH), and 50%/50% (HepG2 with rat S9-mix), respectively. Benchmark dose modeling indicated a higher relative in vitro comet assay response for NFluo compared to NDMA in all cell systems. In conclusion, the in vitro comet assay represents a sensitive and/or specific tool for complementing regulatory in vitro tests in prediction of mutagenic NAs. However, further optimization work is needed, using expanded training sets of compounds and thorough validation of liver cell models, before the in vitro comet assay could be incorporated in the standard battery for genotoxicity testing.

**Supplementary Information:**

The online version contains supplementary material available at https://doi.org/10.1007/s00204-026-04457-1.

## Introduction

*N*-nitrosamines (NAs) are classified as compounds of high concern, due to their genotoxic and carcinogenic properties, with several substances exhibiting high carcinogenic potency in animal models, including rodents (Cheeseman et al. 1999; European Medicines Agency 2025; Kroes et al. 2004). Owing to the pronounced mutagenicity and the carcinogenic potential of many NAs, NA impurities in pharmaceuticals are designated as a "cohort of concern" under the ICH M7(R2) guideline, for which the corresponding Threshold of Toxicological Concern (TTC) approach is not considered appropriate (European Medicines Agency 2025). Notably, many NAs are classified as probable or possible human carcinogens by the International Agency for Research on Cancer (IARC) (Preston-Martin 1987).

For most of the carcinogenic NAs, the established mechanisms of action involve metabolic activation primarily via cytochrome 450 enzymes (CYPs), and mainly through α-hydroxylation, generating highly reactive, electrophilic intermediates, which can ultimately form covalent DNA adducts. If these adducts are not adequately repaired, they can induce DNA replication errors, DNA strand breaks, and mutations, eventually causing tumor development (Fahrer and Christmann 2023; Guttenplan 1987; Li and Hecht 2022a).

Risk assessment of NA impurities in pharmaceuticals remains highly challenging, due to significant data and knowledge gaps. For *N*-Nitrosamine Drug Substance-Related Impurities (NDSRIs), compound-specific carcinogenicity data are mostly unavailable, thus hindering reliable estimation of their carcinogenic potency and the derivation of acceptable intake (AI) limits (Kruhlak et al. 2024; Nudelman et al. 2023). Even for well-characterized small dialkyl NAs, the available carcinogenicity studies may be outdated and did not meet current regulatory standards, thereby limiting their relevance in modern risk assessment (Li et al. 2022; Li and Hecht 2022b).

Under the ICH M7(R2) guideline, the mutagenic potential of impurities is initially assessed using two complementary in silico (Q)SAR models, i.e., one expert rule-based and one statistical-based model to predict the outcome of bacterial reverse mutation tests, such as the Ames test. Alerts for potential bacterial mutagenicity of the impurity may be overruled by a negative OECD TG471-compliant Ames test (International Council for Harmonisation 2023). While effective for multiple chemical classes, this approach is largely inadequate for NAs. The scarcity of high-quality training data, particularly due to structural diversity of NDSRIs, limits predictive accuracy of these models (Kruhlak et al. 2024). Furthermore, experimental OECD TG471-compliant mutagenicity testing requires NA-specific adaptation of the standard protocols (Organisation for Economic Co-operation and Development 2020), as the sensitivity of the standard OECD TG471 Ames test has been questioned for NAs by regulatory agencies (European Medicines Agency 2025; U.S. Food & Drug Administration 2023). Inconsistent results have been reported for several NAs, most likely due to inadequate metabolic activation by the used exogenous metabolic activation systems (Tennant et al. 2023).

To address the limitations of the standard Ames protocol for mutagenicity testing of NAs, an enhanced Ames test (EAT) protocol was developed to increase the sensitivity for testing of NAs (European Medicines Agency 2024). The EAT protocol includes, amongst others, the use of hamster-derived S9-fraction for preparation of S9-mix, as it contains higher levels of certain CYPs relevant for metabolic activation of NAs, compared to the standard rat S9-fraction. While high sensitivity has been reported for the EAT to detect mutagenic NAs, low specificity remains an issue (Thomas et al. 2024).

Consequently, there is increasing interest in the complementary use of genotoxicity/mutagenicity assays based on metabolically competent mammalian cell models, which may offer greater physiological relevance as well as sensitivity and specificity for NAs.

In this context, the in vitro alkaline single cell gel electrophoresis assay (SCGE or comet assay) has gained some attention as a potentially valuable, and complementary tool to the classical Ames test and EAT for predicting genotoxicity of NAs. The alkaline comet assay, originally developed by Singh et al. (1988), is a well-known test to identify DNA-damaging agents in cultured mammalian cells and to characterize various types of DNA damage, including DNA single-strand breaks, DNA double-strand breaks and alkali-labile sites (Ostling and Johanson 1984; Singh et al. 1988). However, the comet assay is generally regarded as an indicator test only, as it cannot detect heritable DNA alterations, such as DNA mutations. Therefore, a positive comet assay result primarily classifies compounds as DNA-damaging but does not necessarily indicate mutagenicity or carcinogenicity. When performed in vivo (Committee et al. 2017; Lambert et al. 2005; OECD 2016; Sasaki et al. 2000), several studies have demonstrated good concordance of the assay with the in vivo transgenic rodent (TGR) somatic mutation assay and also high sensitivity of 92.1% (35 out of 38 chemicals correctly identified) for detecting human IARC group 1 and Group 2A carcinogens, comparable to the TGR assay with 90.3% (Kirkland et al. 2019; Organisation for Economic Co-operation and Development 2022; Zeller et al. 2018). Due to the evidently good predictivity of the in vivo alkaline comet assay, it seemed worthwhile to also evaluate the in vitro alkaline comet assay regarding its potential to detect mutagenic/carcinogenic NAs, when performed with relevant, metabolically competent mammalian tissue or cell types.

In humans, the liver is considered the most sensitive target organ for many orally ingested NAs and exhibits the highest metabolic competence in the body (Camus et al. 1993; Yamazaki et al. 1992). Therefore, liver cells represent a potentially meaningful cell model for in vitro comet assay testing of NAs. An ideal hepatic in vitro model should exhibit sufficient CYP activities, necessary for metabolic activation of NAs. In this regard precision-cut human liver slices (PCLiS), incubated in vitro, might represent a valuable ex vivo liver model for NA testing, due to preservation of both cellular composition, the three-dimensional architecture, and liver-specific functions of the donor liver. Other promising in vitro liver cell models with high metabolic competence include primary human hepatocytes (PHH) and primary rat hepatocytes (PRH). PHH and PRH not only preserve the typical cuboidal cell morphology and metabolic activity in early culture phases but also express numerous influx and efflux transporters for the uptake and elimination of substances and their metabolites (Gomez-Lechon et al. 2003, 2014; Gómez-Lechón et al. 2008; Gupta et al. 2021). Nevertheless, there are some drawbacks of PHH, such as poor availability, donor variability, high procurement costs, and limited culture duration, as they can rapidly lose their liver-specific metabolic competence in vitro. Therefore, alternative hepatocyte models based on cancer cell lines, such as human hepatoblastoma HepG2 cells, have been developed to provide more accessible and scalable systems for in vitro studies. However, HepG2 cells were shown to lack relevant CYP activities necessary for xenobiotic metabolism (Gerets et al. 2012) and are thus in need of exogenous metabolic activation systems, such as supplementation with liver S9-mix or stable overexpression of specific enzymes (Steinbrecht et al. 2019).

Concentration–response data generated from in vitro comet assays can be quantitatively evaluated using benchmark dose (BMD) modeling. BMD modeling is considered well-suited for assessing relative potency and calculating point-of-departure metrics, and it is readily accessible through open-source software (Beal et al. 2023; Heflich et al. 2020; Johnson et al. 2021; MacGregor et al. 2015a, 2015b). Incorporating BMD modeling into the interpretation of in vitro genotoxicity data is in line with modern quantitative risk assessment approaches. The BMD approach employs non-linear regression analysis to establish a BMD level, defined by a predefined response increase relative to the respective negative control. A significant advantage of this method, when compared to other in vitro-derived effect concentrations, such as the minimum effect concentration (MEC) or the lowest observed effect concentration (LOEC), is that BMD modeling utilizes all data contained in a concentration–response curve and accounts for variability in control values. In addition, the uncertainty and precision of the BMD value at the predefined response increase can be established using the two-sided 90% BMD confidence intervals (BMD CI), represented by the upper (BMDU) and lower (BMDL) CI  (White et al. 2019, Slob et al. 2014).

In the present study, we evaluated the in vitro alkaline comet assay with liver cell models as a potential complementary approach to the standard Ames test for predicting the genotoxic potential of NAs. A panel of ten different NAs was investigated, comprising compounds with well-characterized carcinogenicity and mutagenicity profiles (serving as positive and negative controls) as well as NAs with uncertain or unknown genotoxicity, including selected NDSRIs. The study also aimed to assess the performance of different liver cell models in the in vitro comet assay with NAs and to identify the most sensitive and metabolically competent cell system for detecting DNA-damaging effects of NAs.

## Material and methods

### Literature search

To compile available in vitro and in vivo comet assay data on NAs we conducted a literature search focusing on primary publications. This literature search served as an aid in developing optimized study protocols and selecting compounds for the in vitro alkaline comet assay with liver models, including appropriate NA reference compounds. The keywords used were “comet”, “nitrosamine”, and “in vitro” or “in vivo”. The reference sources comprised Science Direct, PubMed, Scopus, and Web of Science.

The comet assay data from the identified publications were subsequently entered into a Microsoft Access database. Data input records were based upon studies, where an experiment with a tissue model or cell type, and an NA was defined as a single study. A single publication, therefore, may contain multiple studies, when the publication presents comet assay data using different tissue models or cell types, and/or different NAs.

### Selection of *N*-nitrosamines

Based on the literature search described above and certain other databases (Table 1), a panel of 10 different NAs was selected at study start in 2022, including some NDSRIs.

**Table 1:** NAs tested in the in vitro alkaline comet assays with liver models based on the literature search in 2022. Ames test data was retrieved from the 2021 Leadscope Genetox SAR database including the CPDB database and FDA Rodent Carcinogenicity database

Compounds	Short name	CAS No	Provider	Ames data	Cancer data	NDSRI
*N*-nitrosodiethanolamine	NDELA	1116–54-7	Enamine*	pos	pos	NO
*N*-nitrosodesloratadine	NND	1246819–22-6	Enamine	ND^1^	ND	YES
*N*-nitrosofluoxetine	NFluo	150494–06-7	Enamine	ND^2^	ND	YES
*N*-nitrosonornicotine (2S)	S-NNN	16543–55-8	Alfa chemistry	pos	pos	NO
Methyl-t-butylnitrosamine	NMtBu	2504–18-9	Enamine	neg	neg	NO
*N*-nitrosofolic acid	NFA	29291–35-8	Selvita	ND^3^	ND	YES
*N*-Methyl-N-nitroso-2-propanamine	NMIPA	30533–08-5	Enamine	ND^4^	ND	NO
*N*-nitrosomethylaniline	NMA	614–00-6	Enamine	pos	pos	NO
*N*-nitrosodimethylamine	NDMA	62–75-9	Enamine*	pos	pos	NO
*N*-nitrosoproline	NPro	7519–36-0	Enamine	neg	neg	NO

ND = no data; neg = negative; pos = positive; *1 g kindly provided by GSK plc.; ^1^recent unpublished, conflicting Ames test results;

^2^ tested positive (Heflich et al. 2024; Jolly et al. 2024); ^3^ tested negative (Heflich et al. 2024); ^4^ tested positive in 2025 (Thomas et al. 2025a)

The compound panel comprised both small and structurally complex NAs as well as NAs with known positive and negative outcome in the Ames test and in carcinogenicity studies, along with NAs with respective data gaps. NDMA, *N*-nitrosodiethanolamine (NDELA), *N*-nitrosonornicotine (2S) (S-NNN), and *N*-nitrosomethylaniline (NMA) were chosen as known mutagenic and carcinogenic compounds (Table 1) (Druckrey et al. 1967; Zielenska and Guttenplan 1988), whereas *N*-methyl-t-butylnitrosamine (NMtBu) and *N*-nitrosoproline (NPro) served as reportedly non-mutagenic and non-carcinogenic compounds (Table 1) (Koepke et al. 1985; Nixon et al. 1976).

### Isolation, culture and treatment of in vitro liver models

#### Precision-cut liver slices (PCLiS)

Human liver tissue was obtained from a female patient undergoing partial hepatectomy. The patient was informed in advance and gave her written consent for her tissue to be used for research purposes. After resection, liver tissue was stored immediately in Belzer UW ® Cold Storage Solution (Bridge to Life, London, UK) and was processed within 3 h after collection. PCLiS were prepared and cultured according to (Granitzny et al. 2017) with minor modifications. In brief, cylindrical tissue cores with a diameter of 8 mm were produced from tissue pieces using a drill equipped with a coring tool. Liver slices (thickness: 200 –300 µm) were prepared in ice-cold Krebs–Henseleit buffer, pH 7.42, supplemented with 25 mM NaHCO_3_, 25 mM D-glucose, 10 mM 2-(4-(2-Hydroxyethyl)- 1-piperazinyl)-ethanesulfonic acid (HEPES) (all from Merck, Darmstadt, Germany) and saturated with carbogen (95% O_2_, 5% CO_2_) using a Krumdieck tissue slicer (Alabama Research and Development, Munford, AL, USA). Slices were then incubated overnight (1 slice/well) in 1 ml oxygenated William’s E Medium supplemented with 1 × GlutaMax supplement, 25 mM D-glucose and 50 µg/ml gentamicin at 37 °C, using a Certomat CT Plus incubator at a shaking frequency of 80 rpm and continuous carbogen flow. After overnight culture in 12-well plates, PCLiS were transferred to new, compound-containing wells and incubated for 2 h with NAs. Cytotoxicity was analyzed by automatic cell counting using a CASY counting device (OLS, OMNI Life Sciences, Bremen, Germany), CASYton isotonic measurement buffer, CASYcups as measurement vessels, and cell type specific size borders.

#### Primary human hepatocytes (PHH)

A 10-donor pool of cryoplateable human hepatocyte, i.e., LIVERPOOL® Mixed Gender Human Hepatocytes (Lot: SWY) was provided by BioIVT (Belgium) together with lot-specific data on viability (84%, post-thaw viability by trypan blue exclusion), number of viable cells per vial (7.33 × 10^6^), enzyme induction, tetrazolium (3-(4,5-dimethylthiazol-2-yl)-2,5-diphenyltetrazolium bromide (MTT) assay-based viability and confluence 5 days after thawing. Additionally, information on liver donors and metabolic activities was provided. Certificate of analysis demonstrated sufficient activity of all NA-relevant CYPs such as CYP3A4, CYP2D6, CYP1A2, CYP2E1, CYP2C9, CYP2B6, CYP2C19, and CYP2A6. Enzyme activities were re-evaluated 24 h after start of pre-culture. PHHs were cultured according to the provider’s instructions using all recommended components to ensure appropriate cell integrity and optimal culture conditions. After thawing, 2.5 × 10^5^ cells were seeded in 0.5 ml of cell plating medium (CP medium, BioIVT) per well in collagen-coated 24-well plates and cultured for 4 h at 37 °C in a humidified atmosphere with 5% CO_2_. Following cell attachment, the medium was replaced by 0.5 ml of serum-free cell incubation medium (HI medium, BioIVT) per well and pre-cultured overnight at 37 °C in an incubator. At 24 h post-plating, the medium was replaced with 0.5 ml of fresh NA-containing HI medium per well or with HI medium containing technical positive and negative reference items . For comet assay use, the cells were detached using 0.25% Trypsin/0.02% EDTA for 10 min after two washing steps with phosphate-buffered saline (PBS). As an indication for cytotoxicity, cell counts were analyzed using automatic cell counting, as described above for PCLiS.

#### Primary rat hepatocytes (PRH)

Male Wistar rats (Janvier Labs, Le Genest-Saint-Isle, France) were used to isolate PRHs by in situ liver perfusion. Rats were anesthetized with pentobarbital, followed by hepatocyte isolation using a two-step EGTA/collagenase-perfusion protocol, as described previously (Carlsson and Fahrer 2023). Cells were used for pre-culture and subsequent NA testing only, if the viability, as determined by trypan blue exclusion, exceeded 85%. For cyto- and genotoxicity testing, isolated hepatocytes (2 × 10^5^ per well) were seeded in 0.5 ml of Dulbecco’s Modified Eagle Medium (DMEM) with low glucose (Thermo Fisher Scientific, Waltham MA, USA) supplemented with 10% fetal calf serum (FCS, Pan-Biotech, Aidenbach, Germany) and 1% Penicillin/Streptomycin solution (Thermo Fisher Scientific, Waltham MA, USA) per well on rat tail collagen-coated 24-well plates at 37 °C in a humidified atmosphere with 5% CO_2._ After a 3 h attachment period, the medium was replaced by 0.5 ml of fresh medium per well, containing the NAs of interest or the positive and negative controls. To estimate cellular sensitivity and for concentration-range finding for the in vitro comet assay, PRHs were exposed for 24 h to seven concentrations of the respective NAs, including a solvent control. Viability was subsequently assessed using the Alamar Blue assay, which is based on resazurin reduction, as previously described (Carlsson et al. 2022).

#### HepG2 cells

Human hepatoblastoma HepG2 cells were purchased from the Leibniz Institute DSMZ-German Collection of Microorganisms and Cell Cultures GmbH (Germany) and cultured in DMEM (PAN-Biotech, Germany), supplemented with 10% fetal calf serum (FCS; PAN-Biotech, Germany) and 0.01% gentamicin (Invitrogen, USA) at 37 °C in a humidified atmosphere with 5% CO_2_. After cell expansion, cells were checked for and found free of mycoplasma contamination. A working batch was prepared from the original cell stock by one laboratory. Cells were subsequently characterized regarding morphology, population doubling time, and chromosome number before being provided to all relevant laboratories. For the comet assay experiments, cells were seeded at a density of 2 × 10^5^ cells/ml per well in 24-well plates and cultured for 24 h prior to treatment. After pre-culture, the medium was replaced by 1 ml of fresh culture medium supplemented with the NAs of interest or the respective technical positive and negative reference items. After treatment (see below), acute cytotoxicity was estimated by automatic cell counting using a CASY counting device, as describedfor PCLiS.

For MTT assays, 5 × 10^3^ HepG2 cells were plated per well of a 96-well plate and treated as described below. Medium was replaced by MTT solution (5 mg/mL in PBS) and incubated for 1 h at 37 °C. MTT solution was removed, formazan crystals were dissolved in dimethyl sulfoxide (DMSO), and absorbance was measured with a Tecan^®^ Microplate Reader at 590 nm.

### Treatment of in vitro liver models

After the cell model specific pre-culture periods, cells were exposed for 2 h (PCLiS, PHH, PRH, and HepG2 cells supplemented with hamster S9-mix) or 4 h (HepG2 cells with rat S9-mix) to the different NAs in increasing concentrations between 0.005 and 10 mM, as indicated. HepG2 cells were incubated with the different NAs in the presence of rat or hamster S9-mix as exogenous metabolizing system, due to insufficient endogenous CYP activities. Respective post-mitochondrial fraction (S9-fraction) was obtained from ICCR-Roßdorf GmbH (Germany). The S9-fractions were prepared from rat liver, which was induced by phenobarbital/β-naphthoflavone, or from non-induced hamster liver. Both protein fractions were stored in liquid nitrogen until use. For this study, S9-fractions with Lot No. 120522 (rat; protein content: 31 mg/ml S9-fraction) and Lot No. 200422 (Syrian hamster; protein content: 31.9 mg/ml S9-fraction) were used, both shown in pre-experiments to exhibit sufficient enzyme activities. For experiments, S9-mix (co-factor-supplemented S9-fraction) was prepared immediately prior to use and was kept on ice until use (Ames et al. 1975; Maron and Ames 1983). S9-mix consisted of 10% (v/v) non-induced hamster or induced rat liver S9-fraction (1 volume S9) and 9 volumes of the respective co-factors (8 mM MgCl_2_, 33 mM KCl, 5 mM glucose-6-phosphate, 4 mM NADP) in sodium orthophosphate buffer (100 mM, pH 7.4). The final concentration of the S9-fraction in the incubation media corresponded to 0.6 mg microsome protein/ml. Water-soluble NAs, such as NDMA, NDELA, and NFA were directly dissolved in the respective cell culture medium, whereas water-insoluble NAs were dissolved in DMSO or ethanol (NND, due to low solubility in DMSO). The NA-solvent mixture was finally diluted in cell culture medium. Final solvent concentration (DMSO or ethanol) did not exceed 0.5% (v/v). As direct technical positive controls for DNA strand break induction, the liver models were treated for 1 h with ethyl methanesulfonate (EMS; PHH, HepG2 cells) or methyl methanesulfonate (MMS; PRH). Cultures treated for 2 h (PHH, PRH, HepG2 cells with hamster S9-mix) or 4 h (HepG2 cells with rat S9-mix) with 5 mM NDMA served as positive control for sufficient metabolic activity.

### Testing of metabolic competence of cells and S9-fractions

PRH and PHH, HepG2 cells, and the rat and hamster S9-fractions were tested for their metabolic competence to ensure metabolic activation of the tested NAs into reactive metabolites by CYPs. To estimate CYP activities in the S9-fractions, 183 µL of PBS (final composition: 10 mM disodium hydrogen phosphate, 1.8 mM potassium dihydrogen phosphate, 137 mM sodium chloride, 2.7 mM potassium chloride, 5 mM magnesium chloride; pH 7.4) was mixed with 5 µL of the respective S9-fractions (20 mg protein/mL), and 2 µL substrate concentrate. The chosen substrates were selective for the respective human CYP isoforms. After 5 min of pre-incubation at 37 °C, 10 µL of 20 mM nicotinamide adenine dinucleotide phosphate (NADPH) tetrasodium salt in PBS was added as cofactor to initiate the reaction, followed by incubation for a further 60 min at a shaking frequency of 550 rpm. All CYP-specific test substrates were dissolved in DMSO and added in CYP-specific concentrations, resulting in a final DMSO concentration of 0.09% (v/v) in the respective incubation media (Table 2).

**Table 2:** CYP isoform-specific substrates with concentrations for testing metabolic competence

CYP isoforms (human)	Orthologue(s) (rat)*	Substrates with concentrations and references	Metabolite
CYP3A4	CYP3A9	Midazolam (100 µM)	6- & alpha-Hydroxymidazolam
CYP2D6	CYP2D3	Dextromethorphan (5 µM)	Dextrorphan
CYP1A2	CYP1A2	Phenacetine (200 µM)	Acetaminophen
CYP2E1	CYP2E1	Chlorzoxazone (120 µM)	6-Hydroxychlorzoxazone & Zopiclone-N-oxide
CYP2C9	CYP2C12	Tolbutamide (80 µM)	4-Hydroxytolbutamide
CYP2B6	CYP2B1/2B2	Bupropion (20 µM)	Hydroxybupropion
CYP2C19	CYP2C13/2C5	S-Mephenytoin (100 µM)	4-Hydroxymephenytoin
CYP2A6	CYP2A1/2A2	Nicotine (60 µM)	Cotinine/Norcotinine

^*^Except CYP2A6 according to (Hammer et al. 2021)

For testing of cell models, 2 × 10^5^ cells were plated per well of 24-well plates and pre-cultured overnight (PHH) or for 3 h (PRH). Cultures were then washed twice with 1 mL of the respective prewarmed cell culture medium before adding 1 mL cell culture medium containing the same substrate concentrations as for S9-fraction testing. The incubation time, buffer, and conditions were chosen to correspond as closely as possible to the final in vitro comet assay protocols. All analytical measurements were performed by Liquid Chromatography-Mass Spectrometry (LC–MS) using a Shimadazu Nexera® UPLC coupled to a SCIEX QTRAP^®^ 6500 Triple Quad mass spectrometer. The measurement of one sample spanned a total of 12 min. Gradient elution was performed starting at a mobile phase composition of 99% solvent A and 1% solvent B, where solvent A consisted of 5 mM ammonium acetate with 0.1% acetic acid adjusted to pH 3.5, and solvent B was acetonitrile. Over a period of 9.5 min, the proportion of solvent B was linearly increased to 100%. This step was followed by a 2.5-min re-equilibration phase under the initial conditions. The chromatographic separation was carried out for each analytical measurement to assess metabolic competence, using an Agilent Poroshell 120^®^ RP18 column (100 × 3 mm, 2.7 µm particle size). The final ion transitions, which were integrated in multiple reaction monitoring (MRM) experiments for semi-quantification of substrate and compound, are shown in Table S4.

To further confirm metabolic competence in selected liver cell models (PHH, HepG2 cells), *CYP* mRNA expression was investigated by quantitative polymerase chain reaction (qRT-PCR). Total RNA of 10^7^ cells was isolated using the RNeasy mini kit (Qiagen), according to the manufacturer’s protocol. RNA was quantified and checked for quality using a Nanodrop® device. RNA ratios A_260_/A_280_: 1.8–2.2, and A_260_/A_230_: > 1.7 were accepted for further analysis. Next, cDNA was synthesized by reverse transcription using the QuantiTect^®^ Reverse Transcription Kit from Qiagen according to the manufacturer’s instructions, and qRT-PCR was finally performed using a QuantiTect® SYBR^®^ Green PCR Kit and QuantiTect Primer Assays (Qiagen) on a LightCycler 480^®^ instrument (Roche). For data analysis, *CYP* mRNA was quantified and normalized to the reference gene *glyceraldehyde-3-phosphate dehydrogenase (GAPDH)* using the DCt method. Reference total RNA from human liver was obtained from Invitrogen^®^.

### In vitro alkaline comet assay

The in vitro alkaline comet assay was generally performed based on the original protocol of Singh et al. (1988) and harmonized as close as possible between the different laboratories and cell types. For the comet assay, glass slides with one roughened surface were pre-coated with two layers of normal melting agarose. In the case of the liver cell models, treated cells were washed using PBS and detached from the well plates using a trypsinization step. Cell suspensions were then centrifuged at 106 × g for 5 min. The cell pellets were subsequently resuspended in 0.75% low melting point agarose and pipetted quickly onto the agarose pre-coated slides. Afterwards, another layer of low melting agarose was added. Cell lysis was performed by immersing the slides in lysis buffer (2.5 M NaCl, 100 mM Na_2_EDTA, 10 mM Tris Base, 1% Triton X-100, 10% DMSO, pH 10) at 4 °C for 1 h or overnight. After a washing step to remove the lysis buffer, slides were placed in the electrophoresis chamber, DNA was unwound for 20 min in 4 °C cold electrophoresis buffer (1 mM Na_2_EDTA and 300 mM NaOH, pH > 13) and electrophoresis was subsequently performed at 0.73 V/cm and 300 mA for 20 min. Slides were then neutralized with 0.4 M Tris–HCl, before being subjected to DNA staining using laboratory-specific staining protocols. 50–150 nuclei per slide and 3 slides per treatment were analyzed using Comet Assay IV systems (Perceptive Instruments Ltd., now Instem). Data were expressed as tail intensity (TI).

For preparation of single cell suspension from PCLiS, liver slices were transferred to 1.5 ml reaction cups containing cell culture medium, followed by a homogenization step using 10 rotations of an appropriate micropestle with moderate pressure. Bigger tissue pieces were eliminated before centrifugation at 106 × g. All subsequent steps were performed as for the liver cell models.

### Statistical analysis

In the in vitro alkaline comet assay, significant differences between control and treated cells were evaluated using the unpaired Welch’s *t*-test, one-tailed (PCLiS, PHH and HepG2 cells), as equal variance was not assumed, or paired *t*-test, one-tailed (PRH, due to variability of each rat), using TIBCO’s Statistica (StatSoft GmbH, Germany). When pre-processing the data, 0.001 was added to all individual single cell TI values to get rid of zero values and these transformed data were converted to logarithms with a base of 10. The median values of the transformed raw data were then calculated per slide, and three independent slides per treatment from three independent wells or three independent experiments were subjected to the respective statistical analyses. Differences from vehicle control were considered statistically significant at a *p*-value ≤ 0.05. A positive result was defined as having a statistically significant difference in at least one concentration, compared to the concurrent vehicle control, along with an at least twofold increase in TI. The means of the raw data from cytotoxicity measurements were subjected to two-sided unpaired Welch’s *t*-test (PHH and HepG2 cells) or two-sided paired *t*-test (PRH). Based on the ICH S2 (R1) guidance document (International Council for Harmonisation, 2011) and White et al.(2020) relevant cytotoxicity was postulated at a minimum of 50% reduction in viability, as compared to the respective vehicle control.

To calculate the sensitivity (true positive rate) and specificity (true negative rate) of each in vitro liver test system for detection of carcinogenic (Table 3) or in vitro/in vivo mutagenic NAs, existing rodent cancer data or in vitro and in vivo mutagenicity data were used as supposed truth (see Table 1 and 4).

**Table 3:** Sensitivity [%] and specificity [%] of four different liver cell models in detecting reference NAs, calculated based on available cancer data

	PHH	PRH	HepG2 cells + 10% rat S9-mix	HepG2 cells + 10% hamster S9-mix
Sensitivity [%]	50	50	50	100
Specificity [%]	100	100	50	50

**Table 4:** Induction of cytotoxicity and DNA strand breaks by NAs in in vitro liver cell models

Literature and calculated data						PHH		PRH		HepG2 + rat S9		HepG2 + hamster S9	
Name	MW [Da]	AI [ng/d]	CPCA	*Mut*	*TD 50* *Rat* *[mg/kg/day]*	Comet assay [mfc]	Cytotox [%]	Comet assay [mfc]	Cytotox [%]	Comet assay [mfc]	Cytotox [%]	Comet assay [mfc]	Cytotox [%]
S-NNN	177.2	100	4	YES	0.0957	< 1	13.4	1.2 (5 mM)	NO	< 1	32.7	3.5	12.6
NDMA	74.1	96	1	YES	0.0959	12	8.5	3.6	12.0	4.7	3	3.7	8.8
NDELA	134.1	1900	3	YES	3.17	5	22.7	2.2	15.5	2.2 (2 mM)	4.4 (2 mM)	2.4 (5 mM)	0
NMA	136.2	100	2	YES	0.142	1.7 (5 mM)	33.5	1.9 (2.5 mM)	41.0 (2.5 mM)	1.1 (5 mM)	0	2.5	19.7
NFluo	338.3	100	1	YES^*^	ND	6.7 (0.5 mM)	10.4	6.4 (0.5 mM)	18.4*** (0.125 mM)	3.9 (0.5 mM)	14.5	3.1 (0.2 mM)	29.9
NPro	144.1	neg	4	NO	neg	1.8	13.5 (5 mM)	1.4 (5 mM)	73.3	1.7 (5 mM)	0	1.5 (2 mM)	0
NMtBu	116.1	1500	5	NO	*(neg)*	< 1	0	1.9	16.7	2.1 (5 mM)	0	2.2 (5 mM)	34.4
NMIPA	102.1	400	3	YES^†^	ND	3.5	14.3 (2 mM)	3.2	26.5 (0.25 M)	1.0	6 (0.5 mM)	3.6	0
NND	310.8	400	3	CD^‡^	ND	1.1 (0.075 mM)	17.6 (0.005 mM)	2.1 (0.175 mM)	7.7*** (0.25 mM)	< 1	10 (0.1 mM)	3.9	26.8
NFA	470.4	NMI	4	NO^§^	ND	1.2	10.8 (2 mM)	1.2 (1 mM)	85.2	< 1	NO	1.8	6.7

^*^(Heflich et al. 2024; Jolly et al. 2024), ^†^(Thomas et al. 2025a), ^‡^recent unpublished, conflicting data (**CD**), ^§^(Heflich et al. 2024)

MW: molecular weight; AI: acceptable intake, retrieved from EMA Appendix 1 (European Medicines Agency 2026); CPCA: “Carcinogenic Potency Categorization Approach” categories; Mut.: bacterial mutagenicity (YES/NO) retrieved from 2021 Leadscope Genetox SAR database including the CPDB database and FDA Rodent Carcinogenicity database and recent publications; TD_50_ Rat: carcinogenic potency retrieved from the Lhasa carcinogenicity database (Gold TD_50_ (Gold et al. 1991); lhasalimited.org); ND: no data; neg: negative; mfc: maximum fold change; NMI: non-mutagenic impurity; Cytotox.: cytotoxicity; red: positive; green: negative; grey: no/conflicting data; mfc is displayed for the highest tested concentration if not otherwise stated in parentheses; *** cytotoxicity at 2 h

$$sensitivity [\%]=\left(\frac{TP}{TP+FN}\right)*100$$

$$specificity \left[\%\right]=\left(\frac{TN}{TN+FP}\right)*100$$

TP: true positive; FN: false negative; TN: true negative; FP: false positive.

### BMD modeling

The obtained comet assay data sets were analyzed by BMD modeling to enable comparative analysis of the tested NAs in the different liver cell models. BMD modeling was carried out using R 4.2.2 (2022–10-31 ucrt) (R Core Team 2022) and the PROAST (version 70.3., RIVM, < https://www.rivm.nl/en/PROAST >) package (RIVM National Institute for Public Health and the Environment) for BMD modeling. Ranking Plots were generated with Python 3.9.12 (< https://www.python.org/ >) as well as seaborn (version 0.11.2) and matplotlib (Version 3.5.1) packages (Hunter 2007; Waskom 2021). For additional plots, ggplot2 (version 3.4.0) and magrittr packages were used (Bache and Wickham 2022; Wickham 2016). BMD modeling was performed for all compound test results which were assessed as positive (3.7). To compare the in vitro DNA damaging activity of different NAs, a predefined “critical effect size” (CES) of 100% was chosen, representing a twofold increase in median-based mean TI of the treated cells over the mean TI value of the respective control. A comparable approach was previously used by Thomas et al. (2024). The corresponding concentration range can be estimated and is referred to as BMD_100_ confidence interval. The lower confidence interval (BMDL_100_), the upper confidence interval (BMDU_100_), their ratio BMD_100_ CI (BMDU_100_/BMDL_100_) as well as the median BMD_100_ was calculated for each NA and for each liver cell system. A model averaging technique (RIVM National Institute for Public Health and the Environment) was used to increase the precision of the analyses by applying 200 bootstrap runs. Concentrations at which precipitation or relevant cytotoxicity (for definition see statistical analysis) were observed, were eliminated prior to modeling to ensure accurate estimation. Data sets with less than three remaining concentrations and those with a positive response yet showed a decreased response and no statistical significance at the highest concentration (compared to negative control), were also excluded from BMD modeling. A prerequisite for the analysis of continuous data within model averaging is a log-normal distribution of the dataset. The distribution per data set was analyzed visually for the four best models of each model family using quantile–quantile plotting (qq-plot). Each data set was first analyzed individually. Covariate BMD analysis was used to increase the precision in the BMD_100_ confidence interval (CIs) calculation (White et al. 2019; Wills et al. 2016b). The covariate approach was applied according to Slob and Setzer (2014) with the assumption that the maximum response (parameter c) and the log-stepness (parameter d) are constant across different datasets. The model fit based on this assumption was checked by visual evaluation of the fitted curves and was only rejected for obvious violations (Slob and Setzer 2014; Thomas et al. 2025b). When assessing the in vitro DNA damaging potency of NAs within a cell type, the NA was used as covariate. Data sets with no trend after model averaging were excluded and not used for further analysis.

In PHH, three independent comet assay experiments were conducted for NDMA with different concentration ranges. All data from these three experiments were combined for BMD analysis. Since the PHH used for the three experiments were from the same 10-donor cell batch, it was reasonable to assume that the data are biologically and statistically comparable and can be combined in a single data pool for concentration–response analysis (U.S. Environmental Protection Agency 2012).

### Ethics

All animal experiments were approved by the government of Rhineland-Palatinate and the Animal Care and Use Committee of the RPTU in Kaiserslautern, Germany (# 23177–07/G22-2–028). The experiments were performed in agreement with the German Federal Law and the guidelines for the protection of animals. Human liver tissue was obtained from a female patient undergoing partial hepatectomy at KRH Siloah hospital (Hannover, Germany). The patient was informed in advance and gave written consent for her tissue to be used for research purposes. Provision of human patient material and experimental protocols were approved by the ethical committee of Hannover Medical School (ref. 9124_BO_K_2020), Hannover, and were in agreement with the German legal requirements. Primary human hepatocytes were obtained from a commercial provider, i.e., BioIVT (Belgium) complying with the relevant ethical standards.

## Results

### Literature search

The literature search on existing comet assay studies testing NAs, revealed 157 in vitro studies involving different primary cells and tumor cell types and 40 in vivo comet assay studies. All these studies were covered by a total of 107 publications, of which 36 in vivo and 57 in vitro studies focused on the liver as target organ. For most of the liver-based in vitro comet assay studies, HepG2 cells served as the model system, irrespective of their low metabolic capacity, followed by human HepaRG hepatoma cells and PHH.

A total of 17 different NAs were investigated in the identified in vitro comet assay studies. As expected, the most studied NAs were NDMA and *N*-nitrosodiethylamine (NDEA), which are the smallest NA from the structural point of few, but among the most potent carcinogenic NAs. Both compounds are classified in category (Cat) 1 of the Carcinogenic Potency Categorization Approach (CPCA), which assigns NAs to one of five categories, ranging from highest (Cat 1) to lowest carcinogenic potency (Cat 5), based on their structural features. Approximately 73% of the in vitro studies identified in this literature search investigated NAs of CPCA Cat 1 (in total 8), indicating that this category was the most extensively studied (see Table S1). These findings highlight the limited data availability regarding comet assay studies considering a higher variety of NAs. Furthermore, for NDMA, the literature search identified 26 studies with a positive outcome in the in vitro comet assay with various in vitro human liver cell models, such as PHH, PRH, HepaRG cells, human induced hepatocytes (HiHEP), and HepG2 cells. There were only six studies in HepG2 cells with NDMA exhibiting a negative outcome. Since NDMA was already extensively investigated in various genotoxicity and mutagenicity assays and showed positive results in different in vitro comet assay studies, this substance was selected as the NA positive control for the present investigations.

### Testing of metabolic competence

For analysis of metabolic competence, the metabolic ratio was used for semi-quantitative calculation. Here, the peak area intensities of the detected metabolites were divided by the sum of the peak areas of the unreacted substrates, and again by the detected metabolites. Due to the large differences between the liver cell models and S9-fractions regarding CYP activities, the metabolic ratios can only be compared semi-quantitatively within the rat and hamster S9-fraction and the liver cell systems.

In contrast to HepG2 cells, both the S9-fractions and primary liver cell models exhibited enzyme activities for the key CYP isoenzymes relevant for the metabolic activation of NAs, more specifically CYP2E1 and CYP2A6. In contrast to PRH, no CYP2E1 activity was observed in PHHs, despite sufficient CYP2E1 activity being reported in the certificate of analysis. It is assumed that the number of cells in the current measurements was too low for sufficient conversion of chlorzoxazone to hydroxychlorzoxazone above the detection limit. HepG2 cells showed no metabolic competence at all. All determined metabolic ratios are listed in Tables S2 and S3. To follow up on the lack of CYP2E1 activity in PHH, we performed qRT-PCRs using total RNA of human liver tissue as positive control. Expression of mRNAs of all CYPs, including CYP2E1, was confirmed in PHH (Fig. S1).

### In vitro alkaline comet assay

**Fig. 1:**
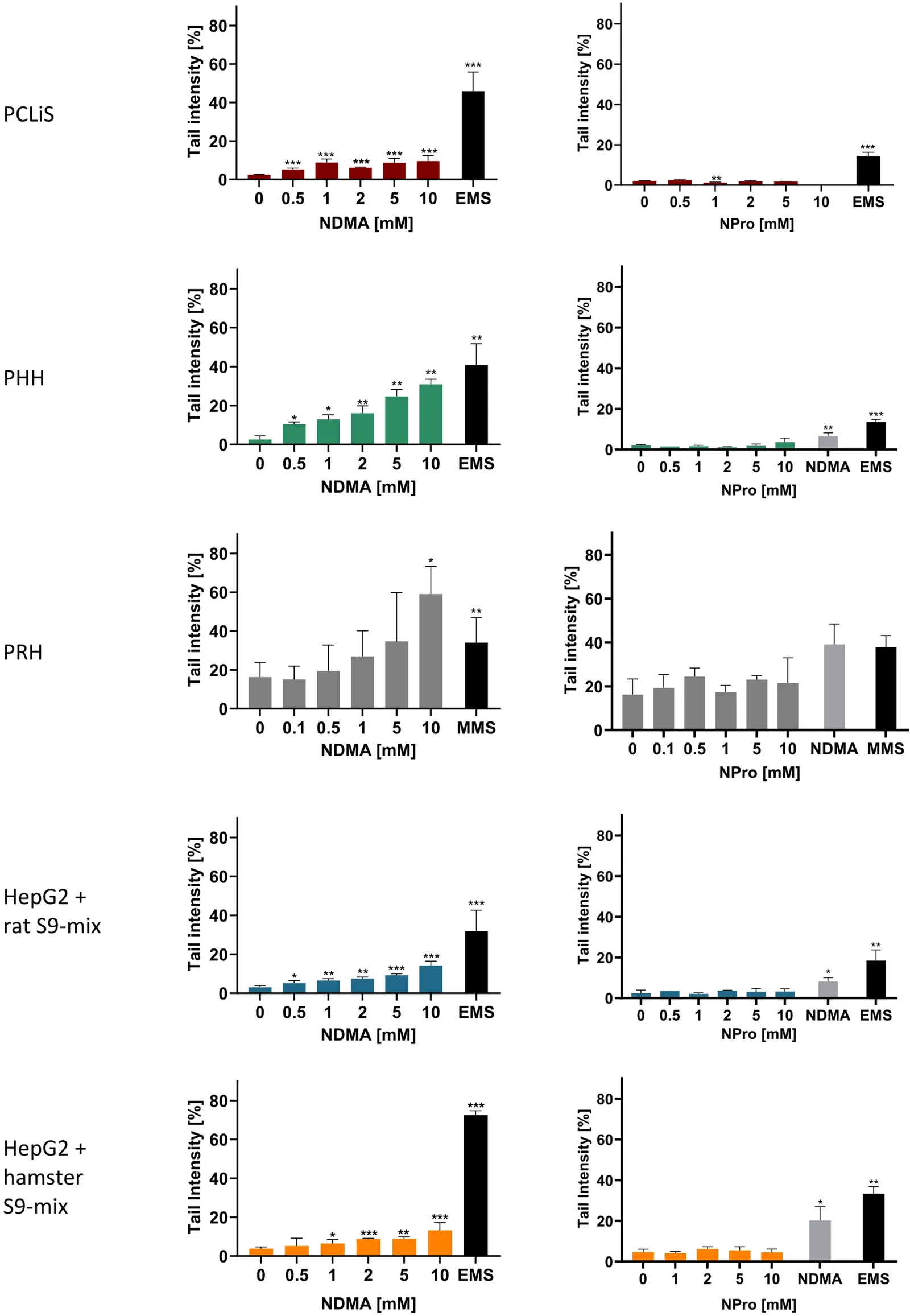
In vitro alkaline comet assays with five different liver cell models incubated for 2 h (PCLiS, PHH, PRH, and HepG2 with hamster S9-mix) or 4 h (HepG2 with rat S9-mix) with increasing concentrations of NDMA (left panel) or NPro (right panel), or the technical positive controls ethyl methanesulfonate (EMS; PHH and HepG2 cells) or methyl methanesulfonate (MMS; PRH) for 1 h. Data represent median-based mean TI ± SD of three biological replicates (three independent cell culture wells for PCLiS, PHH and HepG2 with rat S9-mix, three independent experiments for PRH using cell preparations from three rats on different days, and three independent experiments for HepG2 with hamster S9-mix). * p < 0.05, ** p < 0.01, *** p < 0.001 vs control. Welch’s *t*-test, one-tailed (PHH, PCLiS, and HepG2 cells) or paired *t*-test, one-tailed (PRH) were applied

For protocol optimization and to gain an initial impression of the genotoxic potential of NAs in the selected liver models (PCLiS, PHH, PRH and HepG2 cells in the presence of rat or hamster S9-mix), the test systems were treated with NDMA as one of the most potent mutagenic and carcinogenic NAs and subjected to the in vitro alkaline comet assay. NDMA induced a concentration-dependent increase in DNA strand breaks in all five liver cell models with maximal fold changes (mfcs) of the median-based mean TI of 10.8, 12.0, 3.7, 5.3, and 3.6 over negative control for PCLiS, PHH, PRH, and HepG2 cells with rat or hamster S9-mix, respectively. No signs of cytotoxicity were observed (see Fig. 1, left panel, and Table 4).

To investigate the specificity of the detected NDMA effects, we also tested the known non-carcinogens NPro and NMtBu. NPro was suggested to represent an NA negative control, due to its negative charge at neutral pH, thus preventing its diffusion into cells and subsequent metabolic activation into DNA-reactive metabolites (Chu and Magee 1981; Koepke et al. 1985; Nixon et al. 1976). As expected, NPro did not induce DNA strand breaks in all five liver cell models (Fig. 1, right panel, and Table 4). Cytotoxicity was only observed in PCLiS at 10 mM (65.6%). The second negative control candidate NMtBu did not mediate a significant increase in DNA damage or cytotoxicity in PHH and PRH (Table 4). In HepG2 cells with rat and hamster S9-mix, NMtBu slightly induced DNA strand breaks in a concentration-dependent manner with mfcs of 2.1 and 2.2 at 5 mM, respectively. However, the mean TI decreased below twofold at 10 mM (Fig. S4). NMtBu was thus considered positive for the induction of DNA strand breaks in HepG2 cells with rat and hamster S9-mix, but biological relevance seems questionable. Due to unavailability of appropriate, non-infectious human liver tissue, NMtBu as well as further NAs could not be tested in PCLiS.

Subsequently, three additional NAs with known mutagenicity and carcinogenicity were tested in the in vitro liver cell models, but not in PCLiS. NDELA, classified as carcinogen (IARC 2000), mediated a concentration-dependent increase in mean TI in both PHH, PRH, and HepG2 cells with rat and hamster S9-mix with mfcs of 5.0, 2.2, 2.4, and 2.2, respectively (Table 4). Interestingly, the two carcinogens S-NNN (IARC 2007) and NMA (Zielenska and Guttenplan 1988) induced DNA strand breaks in HepG2 cells with hamster S9-mix only but not in the other liver cell models (Table 4). Based on the available carcinogenicity data of the original NA test panel (S-NNN, NDMA, NDELA, NMA, NPro, and NMtBu) the sensitivity and specificity of the in vitro alkaline comet assay in detection of carcinogenic NAs was calculated for the different liver cell models (Table 3). For PHH and PRH as primary liver cell models sensitivity was 50% and specificity 100%. In contrast, HepG2 cells with hamster S9-mix demonstrated 100% sensitivity for the chosen carcinogenic NAs. However, due to the slightly positive result of NMtBu in this model, specificity amounted to 50%. HepG2 cells with rat S9-mix were identified as the least predictive model with both 50% sensitivity and specificity. Sensitivity and specificity of the in vitro comet assay for prediction of in vitro and/or in vivo mutagenic NAs, including recent Ames test (NFluo, NMIPA, NFA) and in vivo mutagenicity data (NFluo, NFA), were calculated as 67%/100% (PHH, PRH), 50%/67% (HepG2 cells with rat S9-mix) and 100%/67% (HepG2 cells with hamster S9-mix).

**Fig. 2:**
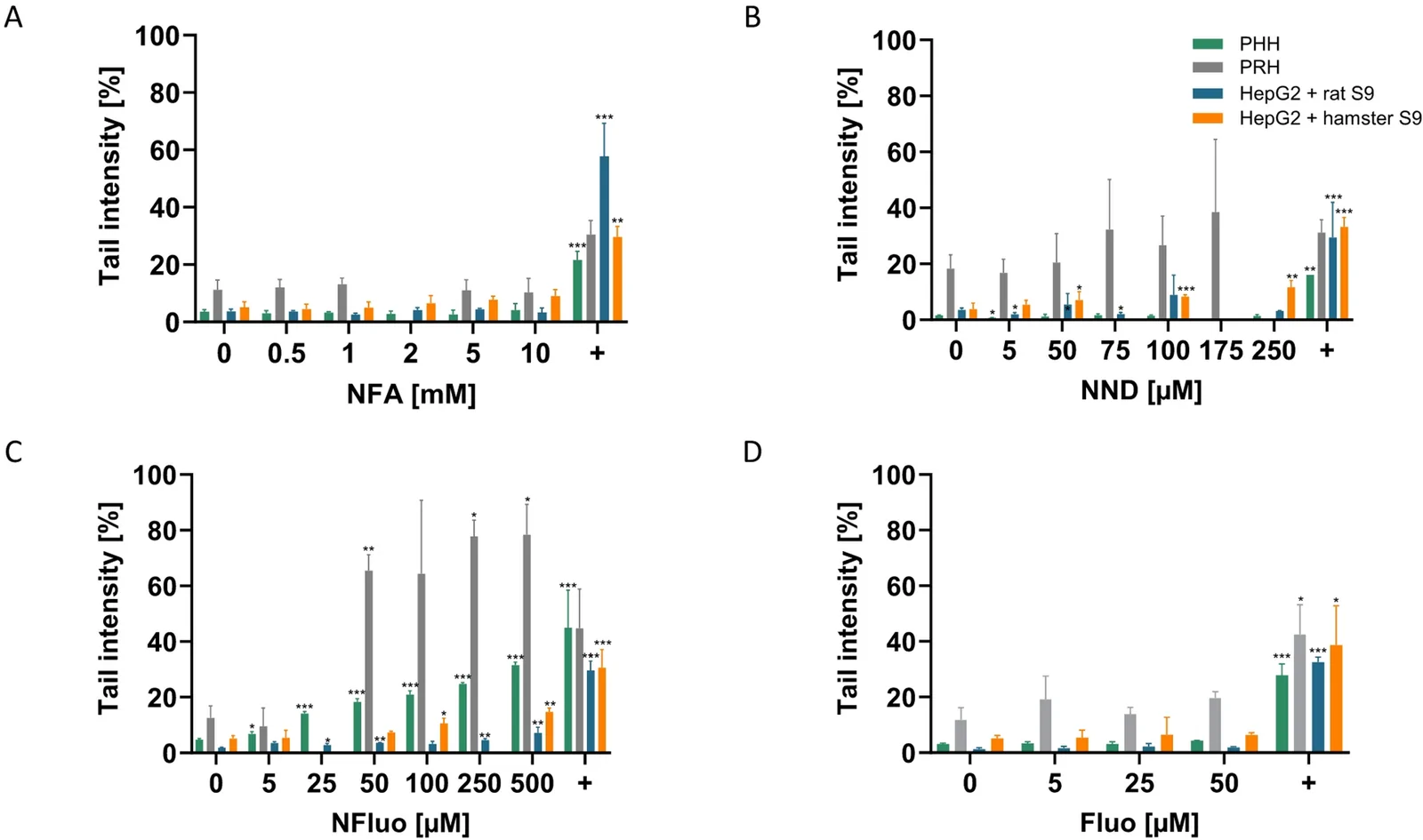
In vitro alkaline comet assays with four different liver cell models incubated for 2 h (PHH [green], PRH [grey], and HepG2 cells with hamster S9-mix [orange]) or 4 h (HepG2 cells with rat S9-mix [blue]) with increasing concentrations of **A** NFA, **B** NND, **C** NFluo, and **D** Fluoxetine (Fluo) or the technical positive controls (+) ethyl methanesulfonate (PHH and HepG2 cells) or methyl methanesulfonate (PRH) for 1 h. Data represent median-based mean TI values ± SD of 3–6 biological replicates (3–6 independent cell culture wells for PHH and HepG2 with rat S9-mix, 3–6 independent cell cultures HepG2 with hamster S9-mix and PRH isolated from 3 animals), respectively. * p < 0.05, ** p < 0.01, *** p < 0.001 versus control. Welch’s *t*-test, one-tailed (PHH and HepG2 cells) or paired *t*-test, one-tailed (PRHs) were applied

We subsequently tested four NAs with unknown carcinogenicity, three of which were NDSRIs. As a non-NDSRI, NMIPA, which in the meantime has been shown to be Ames positive with hamster but not rat S9-mix (Thomas et al. 2025a), caused an increase in TI both in PHH, PRH, and HepG2 cells with hamster S9-mix with mfcs of 3.5, 3.1, and 3.6, respectively, but not in HepG2 with rat S9-mix. NFA, an NDSRI derived from folic acid, and thus from a more complex molecule, has now been classified as non-mutagenic impurity (NMI) by the EMA, as it tested negative in an in vivo mutagenicity study (European Medicines Agency 2026). As expected, NFA induced no DNA strand breaks in all tested liver cell models (Table 4 and Fig. 2A). NND, an NDSRI based on the antihistamine desloratadine with recent unpublished, conflicting Ames test data, demonstrated cytotoxic effects and precipitation at concentrations higher than 0.25 mM. It significantly induced DNA strand breaks in HepG2 cells with hamster S9-mix only, with a mfc of 3.9 at 0.25 mM. Aover twofold increase in mean TI at 175 µM was also observed in PRH but without statistical significance (Table 4 and Fig. 2B).

The known in vitro and in vivo mutagenic NDSRI NFluo (Jolly et al. 2024), derived from the antidepressant fluoxetine, was cytotoxic at concentrations higher than 0.5 mM and induced a significant increase in mean TI in all four liver cell models (Table 4 and Fig. 2C). The highest mfc was observed in PHH with a 6.7-fold increase at 0.5 mM. In contrast, the parent compound fluoxetine (Fluo) did not induce DNA strand breaks in any of the four liver cell models (Fig. 2D), indicating a specific effect of the *N*-nitroso function of the molecule.

### BMD modeling

The in vitro comet assay datasets with positive outcomes were analyzed by fitting concentration–response models using BMD modeling and then ranking the different NAs regarding their relative in vitro comet assay response in the different liver cell models. Since we could only test NDMA with positive outcome in PCLiS, we excluded this liver model from covariate analysis and relative ranking.

When modeling continuous data, the benchmark response is the percent change in mean response relative to the respective controls, which is defined as “critical effect size” (RIVM 2021). The corresponding concentration is referred to as the BMD. In the present analysis, a BMD_100_ CI ratio (BMDU_100_/BMDL_100_) below 10 was considered to be a good estimate, whereas a ratio above 100 indicates high uncertainty of the calculated BMD values (Powley et al. 2024; White et al. 2020, 2019).

**Fig. 3:**
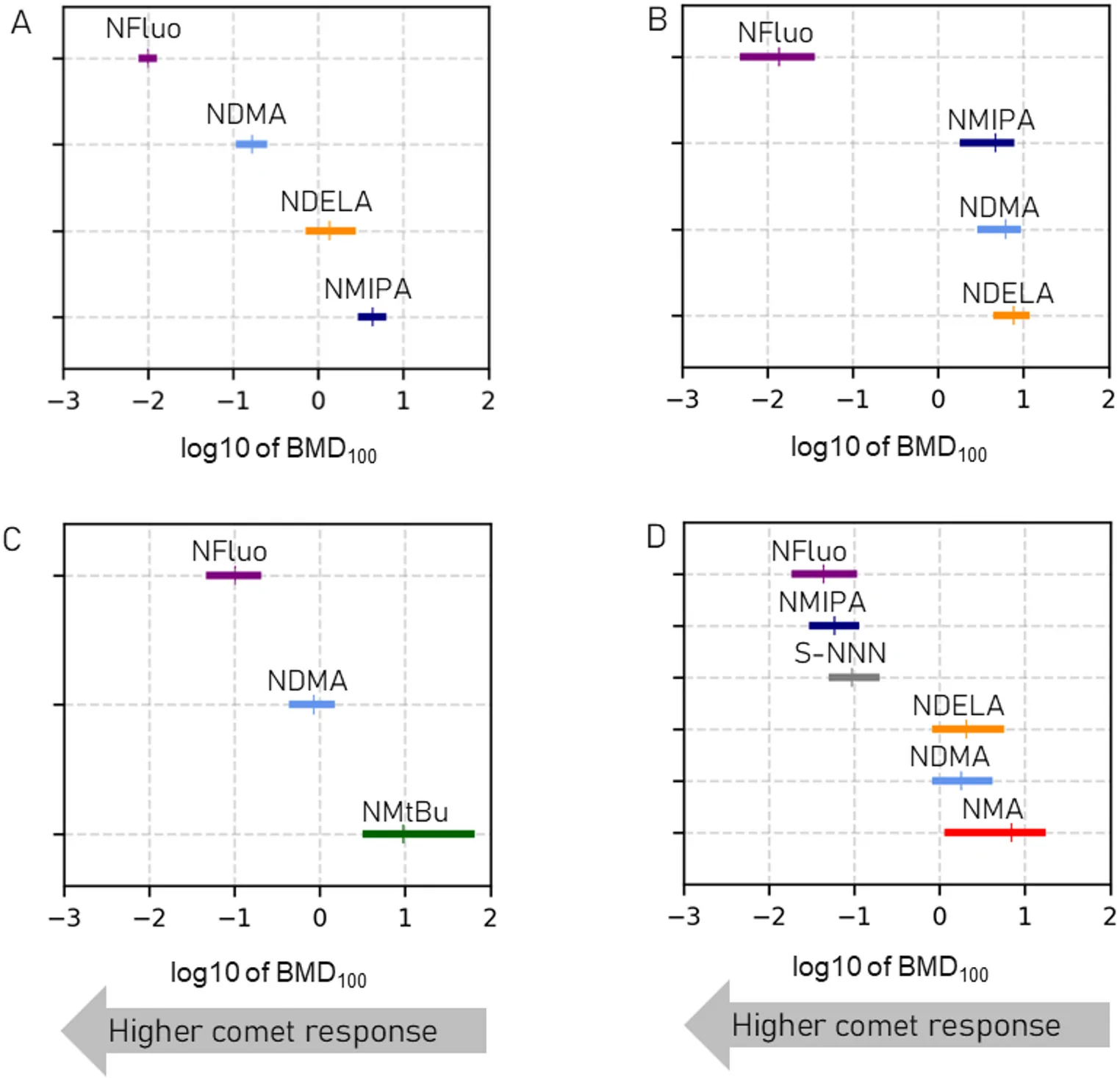
Ranking of DNA-damaging NAs regarding their in vitro comet assay response, based on BMD modeling. The comet assay response of NDMA, NFluo, NDELA, NMIPA, S-NNN, NMtBu and NMA in **A** PHH, **B** PRH, **C** HepG2 cells + rat S9-mix, **D** HepG2 cells + hamster S9-mix was ranked. Data represent log10 of the BMD_100_ values [mM]. The estimated 90% confidence intervals (CI) of the BMD_100_ and the median BMD_100_ (circle) after model averaging and the covariate approach resulting from the calculation of the "critical effect size" of 100 are shown. Left of each line is the BMDL_100_ CI and the right is the BMDU_100_ CI. Analysis was done with PROAST 70.3. In PHH, NDMA was analyzed using three combined experimental datasets. The fitted BMD models and their weighting are presented in the supplements

In both primary liver cell models, NFluo, NDMA, NDELA and NMIPA fulfilled the described criteria for BMD modeling (see 3.8). All derived BMD_100_ values had BMD_100_ CI ratios of < 4 for PHH and < 8 for PRH, indicating good estimates. In PHH, BMD modeling yielded the following comet assay-based response ranking: NFluo > NDMA > NDELA > NMIPA   (Fig. 3A).

In contrast, there were two groups in PRH, i.e., one with NFluo only and one that included an overlap of NDMA, NMIPA and NDELA. NFluo showed a lower concentration range for the BMD_100_ CI than the second group, which was separated by approximately 1.3 log-units, resulting in about 20-fold change in response (Fig. 3B).

In HepG2 cells with rat S9-mix, four NAs tested positive, i.e. NDMA, NFluo, NDELA, and NMtBu. NDELA was positive at one concentration only (2 mM) followed by a negative response (Fig. S4) and was therefore excluded from BMD analysis. There was no overlap of BMD_100_ CI of NFluo and the BMD_100_ CI of NDMA (Fig. 3C), both exhibiting an BMD_100_ CI ratio of < 5. Regarding the in vitro comet assay response, NFluo was thus ranked higher than NDMA followed by NMtBu with a relatively higher BMD_100_ CI ratio of 20.

In HepG2 cells with hamster S9-mix, eight out of ten NAs were tested positive. NMtBu and NND were both excluded from the modeling approach, due to analysis filtering criteria (3.8). The BMD_100_ CI ratios of the remaining NAs, i.e., NDMA, NFluo, NDELA, NMA, and S-NNN showed a BMD_100_ CI ratio of < 8, except for NMA with a BMD_100_ CI ratio of 15.4 (Fig. 3D). For the log_10_ BMD_100_ values calculated from the HepG2 comet assay data sets with hamster S9-mix, two compound groups were evident, one comprising NFluo, NMIPA, and S-NNN with a higher comet assay response and another group comprising NMA, NDMA, and NDELA, ranked lower than group one.

When comparing the four different liver cell systems, notably the NDSRI NFluo was positive in all four liver model systems, and its BMD_100_ CI was consistently located at a lower concentration range than the positive control NDMA. For PHH, PRH, and HepG2 cells with hamster S9-mix, NDELA was consistently in the lower sensitivity part of the compound ranking, whereas no clear trend was present for NMIPA or NDMA.

## Discussion

There is a strong regulatory need for sensitive mutagenicity evaluation of NAs, including NDSRIs, for both approval of new medicines and for assessing safety risks in already approved products. Mutagenicity represents an established surrogate parameter for prediction of the carcinogenic potential, and bacteria-based mutagenicity testing has demonstrated a reasonably good correlation with carcinogenicity, especially for NAs (Bercu et al. 2025). Therefore, in vitro mutagenicity screening of NDSRIs is currently primarily based on Ames testing. However, for promutagens, such as NAs, the Ames test is dependent on the addition of exogenous metabolic activation systems, and there are striking differences in the genomes of bacteria and mammalian cells.

Therefore, the in vitro alkaline comet assay with liver tissue (PCLiS) and liver cell models (PHH, PRH, and HepG2 cells with 10% rat or hamster S9-mix) were evaluated as potential mammalian cell-based tools for prediction of genotoxicity of NAs, as proposed in a recent study (Seo et al. 2025b).

PCLiS, as the only tissue-based model system, demonstrated very promising results in detecting DNA-strand breaks caused by NDMA, and the absence of genotoxicity for the negative control NPro. However, tissue availability represents a major disadvantage of this model. Further experiments using PCLiS from various donors and testing of more NAs would be needed to accurately assess the performance of this human liver model, integrating both different liver cell types and tissue architecture. However, due to limited liver tissue availability and a complex preparation procedure, PCLiS will not represent a standard model for genotoxicity testing of NAs. Nevertheless, PCLiS might serve as a human, close to in vivo reference model for development of less complex and more ubiquitously available cell models, such as 3D HepaRG spheroids (Seo et al. 2024, 2023) after more in depth performance assessment.

For the cell-based liver models, we calculated both sensitivity and specificity for detection of both carcinogenic and in vitro/in vivo mutagenic NAs, based on the outcomes for NAs with available carcinogenicity and in vitro/in vivo mutagenicity data. Four NAs, i.e., NDMA, NDELA, S-NNN, and NMA are known mutagenic carcinogens and served as positive controls. Two known non-carcinogenic NAs, namely NPro and NMtBu, served as negative controls. For NFA, NFluo and NMIPA mutagenicity data were recently published (European Medicines Agency 2026; Jolly et al. 2024; Thomas et al. 2025a). These compounds could therefore be added to the panel of known mutagens (NFluo, NMIPA) and known non-mutagens (NFA).

In the present study, NPro and NFA tested negative in all liver models. In contrast, NMtBu tested negative in PHH and PRH but borderline positive in HepG2 cells with rat and hamster S9-mix. It was previously reported in a computational modelling approach that if DNA base alkylation by tert-butyldiazonium ions occurs, no point mutations may be produced, due to the inability of tert-butylated bases to mis-pair (Salam and Lyngdoh 2021). Here, under certain conditions (e.g., specific cellular systems and CYP activities) tert-butylation of DNA bases by NMtBu may only be moderate, reflected by slightly increased mean TIs in the comet assays, not necessarily leading to mutations. In this case, the comet assay might be over-predictive for mutagenicity and carcinogenicity.

The positive control NDMA was positive in all tested liver models, which was in line with other publications in which primary liver cells and different liver cell lines were treated with NDMA. These include PHH, HepaRG cells, hepatoblastoma HepG2 and HuH6 cells, hepatocellular carcinoma HCC1.2, HCC2, HCC3 and HuH7 cells, as well as rat hepatocytes and microsomes (Ashby et al. 1995; Erkekoglu and Baydar 2010a, 2010b; Frei et al. 2001; Hong et al. 2018; Seo et al. 2019; Uhl et al. 2000; Waldherr et al. 2018; Wilkening et al. 2003; Winter et al. 2008). In agreement with results from a study by Sasaki (2018) using lymphoma cells, NDELA was also positive in all tested liver cells, as did NFluo. However, S-NNN, which was positive in epithelial cells and lymphocytes of oropharyngeal cells (Reiter et al. 2012), was positive only in HepG2 cells with hamster S9-mix, like NMA.

Overall, PHH and PRH exhibited 100% specificity in detecting both carcinogenic and in vitro/in vivo mutagenic NAs correctly. However, sensitivity was only 50% for carcinogenic and 67% for mutagenic NAs, due to their inability to detect the known carcinogens S-NNN and NMA. However, it should be noted that although NNN and NMA can cause liver tumors, liver is not the main target organ for these substances, for which the upper gastrointestinal tract or nasal cavity are the most sensitive sites for carcinogenicity (Hecht et al. 1980; IARC 1987; Kroeger-Koepke et al. 1983). Nevertheless, HepG2 cells with hamster S9-mix were able to detect both S-NNN and NMA and represented the most sensitive test system (sensitivity 100%) but specificity amount to only 50% for known carcinogens and 67% for known mutagens. In contrast, HepG2 cells with rat S9-mix showed substantially lower sensitivity and specificity in comparison to all other liver cell models.

Due to the short-term exposure to NAs (2–4 h) in the present study, the in vitro alkaline comet assay likely detected mainly DNA *N*-alkylation damage and its subsequent repair by base excision repair (BER). DNA *N*-alkylation mainly occurs at the *N*7-position of guanine (e.g., *N*7-methylguanine in case of NDMA), but also at the *N*3-position of adenine (e.g. *N*3-methyladenine in case of NDMA) (Fahrer and Christmann 2023). *N*-alkylated DNA bases such as *N*7-methylguanine and *N*3-methyladenine are recognized by the DNA repair enzyme *N*-alkyladenine DNA glycosylase (AAG), which is also termed* N*-methylpurine DNA glycosylase (MPG) (Engelward et al. 1997). AAG-catalyzed excision of the damaged DNA base give rise to apurinic (AP) sites, which are then converted to DNA single-strand breaks (SSBs) through an incision step, mediated by AP endonuclease 1 (Demple and Sung 2005). These repair intermediates (AP sites and SSBs) can then be detected in the alkaline comet assay (Azqueta and Collins 2013; Ngo et al. 2020).

Notably, DNA *O*-alkylation damage, such as NDMA-induced *O*^6^-methylguanine, cannot be detected by the alkaline comet assay after short incubation periods. *O*^6^-methylguanine adducts trigger DNA strand breaks in a DNA mismatch repair- and replication-dependent manner (Kaina et al. 2010), thus requiring longer incubation periods (≥ 24 h). Furthermore, pharmacological inhibition of the responsible DNA repair enzyme *O*^6^-methylguanine DNA methyltransferase (MGMT) increases the sensitivity of test systems to detect *O*^6^-methylguanine-triggered DNA strand breaks (Carlsson et al. 2025; Kostka et al. 2021; Ramos et al. 2013).

In contrast to the other NAs tested, NFluo, a NDSRI of the selective serotonin uptake inhibitor Fluo, was strongly positive at low micromolar concentrations in all liver cell models tested . This finding is consistent with a very recent publication, revealing that NFluo and other NDSRIs cause DNA methylation adducts and DNA strand breaks in PRH (Vogel et al. 2026). This positive outcome is also in line with recent data on mutagenicity. Jolly et al. (2024) observed both mutagenicity in vitro (Ames test) and in vivo (TGR), and Seo et al. (2025a) demonstrated both in vitro clastogenicity (comet assay, micronucleus test) and mutagenicity (high-fidelity sequencing) in 2D and 3D HepaRG cultures exposed to NFluo for 3 or 14 days. In contrast, the parent compound Fluo caused no induction of DNA strand breaks in all liver cell models, indicating that NFluo-induced DNA damage is NA-specific. However, Fluo showed higher cytotoxicity than NFluo. Therefore, concentrations higher than 50 µM could not be analyzed as there was a complete loss of cells starting at 100 µM.

BMD analysis of the in vitro comet assay data provided insights into the suitability of the assay for predicting carcinogenic/mutagenic NAs. Prior to the analyses shown here, the BMD_50_ was estimated in addition to the BMD_100_ to compare different “critical effect sizes” for ranking of NAs (data not shown). A BMD_100_ approach was previously used by Wills et al. (2016b). Here, the BMD_100_ “critical effect size” proved the most suitable, as shown by the more precise BMD CIs, compared with the respective BMD_50_ CIs. This was due to the BMD_100_ “critical effect sizes” being calculated at a less variable part of the concentration–response curve which, in turn, provides a clearer comparison between the NAs. A value of 100% for the predefined “critical effect sizes” represents a two-fold change, which was also used in the present study to define a biologically relevant effect in the in vitro comet assay thus compensating for data variability and avoiding potentially false-positive results. The choosen BMD_100_ is also in line with a recent publication on the in vitro assessment of NAs using the AMES test (Heflich et al. 2024; Thomas et al. 2024).

The BMD analysis showed that not all CIs for the different NAs were distinguishable from each other within one cell system, based on their relative in vitro comet assay response. A BMD_100_ CI overlap does not necessarily mean that the comet assay response for compounds is the same but indicates that the BMD analysis cannot distinguish between them (Slob 2014; Wills et al. 2016a). In the test system with hamster S9-mix, NMA, NDMA, and NDELA might all be considered equipotent, but with the relatively higher uncertainty (BMD_100_ CI ratio: 15.4) and variability of the NMA data, a final conclusion could not be drawn. NND was positive in only one test system, i.e., HepG2 cells with hamster S9-mix, already at the lowest tested concentration (5 µM). Even with the high uncertainty reflected by the BMD CI width, this NDSRI exhibited a result, which should be analyzed further to rank its mutagenic activity relative to NFluo and NDMA. In both PHH and PRH the in vitro comet assay response was recognizably higher for NFluo than for the other three NAs, but for PRH, NMIPA, NDMA, NDELA might be assumed to be equipotent under the given model assumptions. The results obtained for NDMA and NFluo in the present study contrast with an in vivo rodent TGR mutagenicity study with NFluo which showed a markedly lower mutagenic potency in liver compared to literature data for NDMA and NDEA using BMD modeling (Jolly et al. 2024). This discrepancy was also observed in a previous study by Vogel et al. (2026) showing much higher *N7*-methylguanine and *O*^6^-methylguanine DNA adducts in PRH at 50 µM NFluo shortly after treatment and after 24 h of incubation, as compared to 50 µM NDMA. The potential reasons for this discrepancy were discussed to include toxicokinetic aspects and molecular weight differences.

Lack in NA differentiation and apparently equipotent outcome in PRH, as compared to PHH, might be due to high data variability and highest vehicle control TI values of all tested cell models. This might be attributable to both the cell isolation procedure and the experimental setup used. PRH were treated with NAs on the day of cell isolation 3 h after plating to avoid a decline in metabolic activity and subsequent lack in metabolic conversion of NAs into reactive metabolites at the expense of cell integrity. In contrast, in the case of PHH, the treatment protocol was optimized, based on cellular fitness, resulting in lower vehicle TI values and data variability, however at the expense of metabolic competence. PHH initially demonstrated a loss in membrane integrity when incubated with NAs on the day of thawing, and, therefore, pre-culture for 24 h was introduced into the treatment protocol to avoid artifactual results. However, pre-culture seemed to reduce metabolic competence, as CYP2E1 activity was not detected using a CYP2E1-specific substrate, although mRNA expression was confirmed using qRT-PCR. This was in contrast to reported CYP2E1 activities in the certificate of analysis. A further optimization, based on a compromise between metabolic competence and cellular fitness, may provide higher metabolic activity and more stable background TI values.

Overall, based on our analyses with a limited number of NAs, BMD modeling seems promising for ranking the relative concentration–response of NAs with reference to the in vitro comet assay. However, more data is needed to draw final conclusions on the applicability of the present BMD modeling approach for ranking of the relative genotoxic potency of NAs in the in vitro comet assay.

For the in vitro comet assay, the choice of the preferred cell model depends on the focus of the investigation. Based on the present results, a primary liver cell model should preferably be used to avoid exogenous metabolic activation systems, which are needed in the case of metabolically incompetent cell lines. In primary liver cell models, NAs are activated intracellularly by CYPs after cell entrance. The resulting reactive metabolites have to cross the nuclear membrane only to bind to DNA. For cell models in need of external metabolizing systems such as S9-mix, NAs are already activated extracellularly and the activated/reactive metabolites must cross both cell and nuclear membranes for DNA adduct formation. This could potentially result in a different outcome for cell lines and primary cells, if the original NA, but not the reactive metabolites can cross cell membranes, or if highly reactive metabolites can already react with media or membrane components. The high sensitivity (100% for the known carcinogenic and mutagenic NAs tested here) of HepG2 cells supplemented with hamster S9-mix, as compared to PHH and PRH, in principle might make this system a valuable tool for hazard identification and potential follow up of negative/borderline EAT test results to provide further reassurance for the EAT result. However, the obviously lower specificity of this model might result in a higher potential for irrelevant, positive outcomes and overprediction of NA mutagenicity. The high sensitivity but lower specificity of the HepG2 cell model with hamster S9-mix therefore makes the model useful when a conservative approach to avoid false-negative results is the goal of the test strategy.

Another important factor to consider in the context of appropriate liver cell models is the translation of results to humans and rodents. In this regard, primary liver cell models may be more predictive, as they can cover not only phase I but also phase II metabolism, and thus possible deactivation of certain NAs (Madle et al. 1986). Furthermore, in vitro human hepatocytes were shown to have a comparable metabolism to the in vivo situation (Ponsoda et al. 2001). According to the present results, PHH might be preferred over PRH, as TI variability in the vehicle control was lower and BMD results more consistent in PHHs. However, disadvantages of primary human liver cells consist of their limited availability, their high costs, potential inter-donor or batch-to-batch variability in metabolic competence, and potential rapid decline in metabolic activity, thus making them a suboptimal liver cell model for standard test batteries. Therefore, the use of genetically engineered cells with human CYP overexpression or HepaRG cells with stable metabolic competence, low batch-to-batch differences and the potential to form bile canaliculi and functional tight junctions might represent more valuable, practical alternatives for genotoxicity screening of NAs, as recently reported (Carlsson et al. 2025; Li et al. 2024; Seo et al. 2025b). However, primary cells of different species (Seo et al. 2025b) or PCLiS might aid in confirmation of screening results or serve as reference model in establishment of advanced liver cell models for genotoxicity testing of NAs.

In conclusion, the in vitro comet assay represents a sensitive and/or specific tool for complementing existing regulatory in vitro tests in prediction of mutagenic NAs. This might be useful in cases where identification of responsible metabolites or types of DNA strand breaks is required, or for antibiotics where in vitro bacterial mutagenicity tests are not feasible. Primary cells, particularly PHH, are preferred over tumor cell lines as cell models, due to their metabolic competence and closer similarity to the in vivo environment, however with certain limitations as standard testing models. The use of the in vitro comet assay with liver cell models can already be considered as part of an overall weight of evidence approach, but further optimization work, including expanded training sets of compounds and thorough validation of appropriate and ubiquitously available liver cell models would be required, before it could be incorporated into a standard battery for genotoxicity testing of NAs.

## Supplementary Information

Below is the link to the electronic supplementary material.

Supplementary Material 1

## Data Availability

All data supporting the findings of this study are available within the paper and its Supplementary Information and are also available on request from authors.

## References

[CR1] Organisation for Economic Co-operation and Development (2020) Ninth Addendum to OECD Guidelines for Testing of Chemicals, Section 4, No. 471 Bacterial Reverse Mutation Test. Organisation for Economic Co-operation and Development

[CR2] European Medicines Agency (2026) European Medicines Agency Appendix 1 to Questions and answers for marketing authorisation holders/applicants on the CHMP Opinion for the Article 5 (3) of Regulation (EC) No 726/2004 referral on nitrosamine impurities in human medicinal products. EMA/CHMP/42189/2026/Rev. 12

[CR3] International Council for Harmonisation of Technical Requirements for Pharmaceuticals for Human Use (2023) ICH M7(R2) Guideline on assessment and control of DNA reactive (mutagenic) impurities in pharmaceuticals to limit potential carcinogenic risk.

[CR4] European Medicines Agency (2025) Questions and answers for marketing authorisation holders/applicants on the CHMP Opinion for the Article 5(3) of Regulation (EC) No 726/2004 referral on nitrosamine impurities in human medicinal products. EMA/409815/2020 Rev. 23

[CR5] Ames BN, Mc Cann J, Yamasaki E (1975) Mutation Res 316:347 10.1016/0165-1161(75)90046-1 768755

[CR6] European Medicines Agency (2024) European Medicines Agency Appendix 3 to Questions and answers for marketing authorisation holders/applicants on the CHMP Opinion for the Article 5 (3) of Regulation (EC) No 726/2004 referral on nitrosamine impurities in human medicinal products. Enhanced Ames Test Conditions for N-nitrosamines. EMA/120337/2024

[CR7] Ashby J, Tinwell H, Lefevre PA et al (1995) The single cell gel electrophoresis assay for induced DNA damage (comet assay): measurement of tail length and moment. Mutagenesis 10(2):85–90. 10.1093/mutage/10.2.85 7603334

[CR8] Azqueta A, Collins AR (2013) The essential comet assay: a comprehensive guide to measuring DNA damage and repair. Arch Toxicol 87(6):949–968. 10.1007/s00204-013-1070-0 23685795

[CR9] Bache SM, Wickham H (2022) magrittr: A Forward-Pipe Operator for R. R package version 2.0.3 edn

[CR10] Beal MA, Audebert M, Barton-Maclaren T et al (2023) Quantitative in vitro to in vivo extrapolation of genotoxicity data provides protective estimates of in vivo dose. Environ Mol Mutagen 64(2):105–122. 10.1002/em.22521 36495195

[CR11] Bercu J, Trejo-Martin A, Chen C et al (2025) HESI GTTC ring trial: concordance between Ames and rodent carcinogenicity outcomes for N-nitrosamines (NAs) with rat and hamster metabolic conditions. Regul Toxicol Pharmacol 161:105835. 10.1016/j.yrtph.2025.105835 40311791

[CR12] Camus AM, Geneste O, Honkakoski P et al (1993) High variability of nitrosamine metabolism among individuals: role of cytochromes P450 2A6 and 2E1 in the dealkylation of N-nitrosodimethylamine and N-nitrosodiethylamine in mice and humans. Mol Carcinog 7(4):268–275. 10.1002/mc.2940070410 8352885

[CR13] Carlsson MJ, Fahrer J (2023) Analyzing the Effects of HDAC Inhibitors on DNA Damage and Associated Cytotoxicity in Primary Hepatocytes. Methods in Molecular Biology (Clifton, NJ) 2589:241–252. 10.1007/978-1-0716-2788-4_16 36255629

[CR14] Carlsson MJ, Vollmer AS, Demuth P et al (2022) p53 triggers mitochondrial apoptosis following DNA damage-dependent replication stress by the hepatotoxin methyleugenol. Cell Death Dis 13(11):1009. 10.1038/s41419-022-05446-9 36446765 PMC9708695

[CR15] Carlsson MJ, Herzog N, Felske C et al (2025) The DNA repair protein MGMT protects against the genotoxicity of N-nitrosodimethylamine, but not N-nitrosodiethanolamine and N-nitrosomethylaniline, in human HepG2 liver cells with CYP2E1 expression. Chem Res Toxicol 38(6):1134–1146. 10.1021/acs.chemrestox.5c00133 40390554

[CR16] Cheeseman MA, Machuga EJ, Bailey AB (1999) A tiered approach to threshold of regulation. Food Chem Toxicol 37(4):387–412. 10.1016/S0278-6915(99)00024-1 10418955

[CR17] Chu C, Magee PN (1981) Metabolic fate of nitrosoproline in the rat. Cancer Res 41(9 Pt 1):3653–3657 6167353

[CR18] Committee ES, Hardy A, Benford D et al (2017) Guidance on the risk assessment of substances present in food intended for infants below 16 weeks of age. EFSA J 15(5):e04849. 10.2903/j.efsa.2017.4849 32625502 PMC7010120

[CR19] Demple B, Sung JS (2005) Molecular and biological roles of Ape1 protein in mammalian base excision repair. DNA Repair (Amst) 4(12):1442–1449. 10.1016/j.dnarep.2005.09.004 16199212

[CR20] Druckrey H, Preussmann R, Ivankovic S et al (1967) Organotrope carcinogene Wirkungen bei 65 verschiedenen N-Nitroso-Verbindungen an BD-Ratten [Organotropic carcinogenic effects of 65 various N-nitroso- compounds on BD rats]. Zeitschrift fur Krebsforschung 69(2):103–201 4230610

[CR21] Engelward BP, Weeda G, Wyatt MD et al (1997) Base excision repair deficient mice lacking the Aag alkyladenine DNA glycosylase. Proc Natl Acad Sci U S A 94(24):13087–13092. 10.1073/pnas.94.24.13087 9371804 PMC24267

[CR22] Erkekoglu P, Baydar T (2010a) Effect of allyl isothiocyanate (AITC) in both nitrite- and nitrosamine-induced cell death, production of reactive oxygen species, and DNA damage by the single-cell gel electrophoresis (SCGE): does it have any protective effect on HepG2 cells? Int J Toxicol 29(3):305–312. 10.1177/1091581810366313 20448263

[CR23] Erkekoglu P, Baydar T (2010b) Evaluation of the protective effect of ascorbic acid on nitrite- and nitrosamine-induced cytotoxicity and genotoxicity in human hepatoma line. Toxicol Mech Methods 20(2):45–52. 10.3109/15376510903583711 20100056

[CR25] Fahrer J, Christmann M (2023) DNA alkylation damage by nitrosamines and relevant DNA repair pathways. Int J Mol Sci. 10.3390/ijms24054684 36902118 PMC10003415

[CR26] U.S. Environmental Protection Agency (2012) Benchmark Dose Technical Guidance. In: Forum RA (ed). vol EPA/100/R-12/001. U.S. Environmental Protection Agency,, Washington, DC

[CR27] Frei E, Kuchenmeister F, Gliniorz R et al (2001) N-nitrososdimethylamine is activated in microsomes from hepatocytes to reactive metabolites which damage DNA of non-parenchymal cells in rat liver. Toxicol Lett 123(2–3):227–234. 10.1016/s0378-4274(01)00400-3 11641050

[CR28] Gerets HH, Tilmant K, Gerin B et al (2012) Characterization of primary human hepatocytes, HepG2 cells, and HepaRG cells at the mRNA level and CYP activity in response to inducers and their predictivity for the detection of human hepatotoxins. Cell Biol Toxicol 28(2):69–87. 10.1007/s10565-011-9208-4 22258563 PMC3303072

[CR29] Gold LS, Slone TH, Manley NB et al (1991) The Carcinogenic Potency Database: analyses of 4000 chronic animal cancer experiments published in the general literature and by the U.S. National Cancer Institute/National Toxicology Program. Environ Health Perspect 96:11–15. 10.1289/ehp.919611 1820251 PMC1568255

[CR30] Gomez-Lechon MJ, Donato MT, Castell JV et al (2003) Human hepatocytes as a tool for studying toxicity and drug metabolism. Curr Drug Metab 4(4):292–312. 10.2174/1389200033489424 12871046

[CR31] Gomez-Lechon MJ, Tolosa L, Conde I et al (2014) Competency of different cell models to predict human hepatotoxic drugs. Expert Opin Drug Metab Toxicol 10(11):1553–1568. 10.1517/17425255.2014.967680 25297626

[CR32] Gómez-Lechón MJ, Cj V, Donato MT (2008) An update on metabolism studies using human hepatocytes in primary culture. Expert Opin Drug Metab Toxicol 4(7):837–854. 10.1517/17425255.4.7.837 18624674

[CR33] Granitzny A, Knebel J, Schaudien D et al (2017) Maintenance of high quality rat precision cut liver slices during culture to study hepatotoxic responses: Acetaminophen as a model compound. Toxicol in Vitro 42:200–213. 10.1016/j.tiv.2017.05.001 28476500

[CR34] Gupta R, Schrooders Y, Hauser D et al (2021) Comparing in vitro human liver models to in vivo human liver using RNA-Seq. Arch Toxicol 95(2):573–589. 10.1007/s00204-020-02937-6 33106934 PMC7870774

[CR35] Guttenplan JB (1987) N-nitrosamines: bacterial mutagenesis and in vitro metabolism. Mutat Res 186(2):81–134. 10.1016/0165-1110(87)90026-1 3306361

[CR36] Hammer H, Schmidt F, Marx-Stoelting P et al (2021) Cross-species analysis of hepatic cytochrome P450 and transport protein expression. Arch Toxicol 95(1):117–133. 10.1007/s00204-020-02939-4 33150952 PMC7811513

[CR37] Hecht SS, C-hB C, Ohmori T et al (1980) Comparative Carcinogenicity in F344 Rats of the Tobacco-specific Nitrosamines, N′-Nitrosonornicotine and 4-(N-Methyl-N-nitrosamino)-1-(3-pyridyl)-1-butanone1. Can Res 40(2):298–302 7356512

[CR38] Heflich RH, Johnson GE, Zeller A et al (2020) Mutation as a toxicological endpoint for regulatory decision-making. Environ Mol Mutagen 61(1):34–41. 10.1002/em.22338 31600846

[CR39] Heflich RH, Bishop ME, Mittelstaedt RA et al (2024) Optimizing the detection of N-nitrosamine mutagenicity in the Ames test. Regul Toxicol Pharmacol 153:105709. 10.1016/j.yrtph.2024.105709 39343352 PMC12395388

[CR40] Hong YH, Jeon HL, Ko KY et al (2018) Assessment of the predictive capacity of the optimized in vitro comet assay using HepG2 cells. Mutat Res Genet Toxicol Environ Mutagen 827:59–67. 10.1016/j.mrgentox.2018.01.010 29502738

[CR41] Hunter JD (2007) Matplotlib: a 2D graphics environment. Comput Sci Eng 9(3):90–95

[CR42] IARC (1987) IARC monographs on the evaluation of the evaluation of the carcinogenic risks to humans, vol vol Supplement 7. International Agengy for Research on Cancer, Lyon

[CR43] IARC (2000) Some industrial chemicals - IARC monographs on the evaluation of carcinogenic risks to humans. Some industrial chemicals some industrial chemicals - IARC monographs on the evaluation of carcinogenic risks to humans some industrial chemicals, vol 77. International Agency for Research on Cancer, Lyon, pp 403–438

[CR44] IARC (2007) Smokeless Tobacco and Some Tobacco-specific N-Nitrosamines. IARC Monographs on the Evaluation of Carcinogenic Risks to Humans Volume 89 PMC4781254 18335640

[CR45] Johnson GE, Dobo K, Gollapudi B et al (2021) Permitted daily exposure limits for noteworthy N-nitrosamines. Environ Mol Mutagen 62(5):293–305. 10.1002/em.22446 34089278

[CR46] Jolly RA, Cornwell PD, Noteboom J et al (2024) Estimation of acceptable daily intake values based on modeling and in vivo mutagenicity of NDSRIs of fluoxetine, duloxetine and atomoxetine. Regul Toxicol Pharmacol 152:105672. 10.1016/j.yrtph.2024.105672 38968965

[CR47] Kaina B, Margison GP, Christmann M (2010) Targeting O6-methylguanine-DNA methyltransferase with specific inhibitors as a strategy in cancer therapy. Cell Mol Life Sci 67(21):3663–3681. 10.1007/s00018-010-0491-7 20717836 PMC11115711

[CR48] Kirkland D, Levy DD, LeBaron MJ et al (2019) A comparison of transgenic rodent mutation and in vivo comet assay responses for 91 chemicals. Mutat Res Genet Toxicol Environ Mutagen 839:21–35. 10.1016/j.mrgentox.2019.01.007 30744809 PMC6697155

[CR49] Koepke SR, Tondeur Y, Farrelly JG et al (1985) Metabolic studies of 15N-labeled N-nitrosoproline in isolated rat hepatocytes and subcellular fractions. Cancer Lett 27(3):277–283. 10.1016/0304-3835(85)90185-5 4016722

[CR50] Kostka T, Empl MT, Seiwert N et al (2021) Repair of O6-carboxymethylguanine adducts by O6-methylguanine-DNA methyltransferase in human colon epithelial cells. Carcinogenesis 42(8):1110–1118. 10.1093/carcin/bgab049 34115837

[CR51] Kroeger-Koepke MB, Reuber MD, Iype PT et al (1983) The effect of substituents in the aromatic ring on carcinogenicity of N-nitrosomethylaniline in F344 rats. Carcinogenesis 4(2):157–160. 10.1093/carcin/4.2.157 6402317

[CR52] Kroes R, Renwick AG, Cheeseman M et al (2004) Structure-based thresholds of toxicological concern (TTC): guidance for application to substances present at low levels in the diet. Food Chem Toxicol 42(1):65–83. 10.1016/j.fct.2003.08.006 14630131

[CR53] Kruhlak NL, Schmidt M, Froetschl R et al (2024) Determining recommended acceptable intake limits for N-nitrosamine impurities in pharmaceuticals: development and application of the Carcinogenic Potency Categorization Approach (CPCA). Regul Toxicol Pharmacol 150:105640. 10.1016/j.yrtph.2024.105640 38754805

[CR54] Lambert IB, Singer TM, Boucher SE et al (2005) Detailed review of transgenic rodent mutation assays. Mutat Res 590(1–3):1–280. 10.1016/j.mrrev.2005.04.002 16081315

[CR55] Li Y, Hecht SS (2022a) Metabolic activation and DNA interactions of carcinogenic N-Nitrosamines to which humans are commonly exposed. Int J Mol Sci 23(9):4559. 10.3390/ijms23094559 35562949 PMC9105260

[CR56] Li Y, Hecht SS (2022b) Metabolic activation and DNA interactions of carcinogenic N-Nitrosamines to which humans are commonly exposed. Int J Mol Sci. 10.3390/ijms23094559 35562949 PMC9105260

[CR57] Li X, He X, Le Y et al (2022) Genotoxicity evaluation of nitrosamine impurities using human TK6 cells transduced with cytochrome P450s. Arch Toxicol. 10.1007/s00204-022-03347-6 35882637 PMC12392337

[CR58] Li X, Le Y, Guo X et al (2024) Mutagenicity and genotoxicity evaluation of 15 nitrosamine drug substance-related impurities in human TK6 cells. Regul Toxicol Pharmacol 154:105730. 10.1016/j.yrtph.2024.105730 39433234 PMC12477663

[CR59] MacGregor JT, Frotschl R, White PA et al (2015a) IWGT report on quantitative approaches to genotoxicity risk assessment II. Use of point-of-departure (PoD) metrics in defining acceptable exposure limits and assessing human risk. Mutat Res Genet Toxicol Environ Mutagen 783:66–78. 10.1016/j.mrgentox.2014.10.008 25953401

[CR60] MacGregor JT, Frotschl R, White PA et al (2015b) IWGT report on quantitative approaches to genotoxicity risk assessment I. Methods and metrics for defining exposure-response relationships and points of departure (PoDs). Mutat Res Genet Toxicol Environ Mutagen 783:55–65. 10.1016/j.mrgentox.2014.09.011 25953400

[CR61] Madle E, Tiedemann G, Madle S et al (1986) Comparison of S9 mix and hepatocytes as external metabolizing systems in mammalian cell cultures: cytogenetic effects of 7,12-dimethylbenzanthracene and aflatoxin B1. Environ Mutagen 8(3):423–437. 10.1002/em.2860080311 3086074

[CR62] Maron DM, Ames BN (1983) Revised methods for the Salmonella mutagenicity test. Mutat Res Environ Mutagen Relat Subj 113(3–4):173–215. 10.1016/0165-1161(83)90010-9 6341825

[CR63] Ngo LP, Owiti NA, Swartz C et al (2020) Sensitive CometChip assay for screening potentially carcinogenic DNA adducts by trapping DNA repair intermediates. Nucleic Acids Res 48(3):e13. 10.1093/nar/gkz1077 31822921 PMC7026589

[CR64] Nixon JE, Wales JH, Scanlan RA et al (1976) Null carcinogenic effect of large doses of nitrosoproline and nitrosohydroxyproline in Wistar rats. Food Cosmet Toxicol 14(2):133–135. 10.1016/s0015-6264(76)80257-x 178581

[CR65] Organisation for Economic Co-operation and Development (2022) Test No. 488: Transgenic Rodent Somatic and Germ Cell Gene Mutation Assays,

[CR66] Nudelman R, Kocks G, Mouton B et al (2023) The Nitrosamine “saga”: lessons learned from five years of scrutiny. Org Process Res Dev 27(10):1719–1735. 10.1021/acs.oprd.3c00100

[CR67] OECD (2016) GUIDELINE FOR THE TESTING OF CHEMICALS No. 489 In Vivo Mammalian Alkaline Comet Assay

[CR68] Ostling O, Johanson KJ (1984) Microelectrophoretic study of radiation-induced DNA damages in individual mammalian cells. Biochem Biophys Res Commun 123(1):291–298. 10.1016/0006-291x(84)90411-x 6477583

[CR69] Ponsoda X, Pareja E, Gómez-Lechón M-J et al (2001) Drug biotransformation by human hepatocytes. in vitro/in vivo metabolism by cells from the same donor. J Hepatol 34(1):19–25. 10.1016/S0168-8278(00)00085-4 11211902

[CR70] Powley MW, Sobol Z, Johnson GE et al (2024) N-nitrosamine impurity risk assessment in pharmaceuticals: utilizing in vivo mutation relative potency comparison to establish an acceptable intake for NTTP. Regul Toxicol Pharmacol 152:105681. 10.1016/j.yrtph.2024.105681 39067806

[CR71] Preston-Martin S (1987) N-nitroso compounds as a cause of human cancer. IARC Sci Publ 84:477–484 3679426

[CR72] R Core Team (2022) R: a language and environment for statistical computing, vol 4.2.2. R Foundation for Statistical Computing, Vienna, Austria

[CR73] Ramos AA, Pedro DFN, Lima CF et al (2013) Development of a new application of the comet assay to assess levels of O6-methylguanine in genomic DNA (CoMeth). Free Radic Biol Med 60:41–48. 10.1016/j.freeradbiomed.2013.01.028 23391575

[CR74] Reiter M, Baumeister P, Jaiser S et al (2012) DNA repair and mutagen sensitivity of epithelial cells and lymphocytes in oropharyngeal cancer. Oncol Lett 3(1):100–106. 10.3892/ol.2011.417 22740863 PMC3362385

[CR75] RIVM (2021) PROAST Manual version 70.3.

[CR76] RIVM National Institute for Public Health and the Environment PROAST. 70.3 edn

[CR77] Salam T, Lyngdoh RHD (2021) Clues to the non-carcinogenicity of certain N-Nitroso compounds: role of alkylated DNA bases. Biophys Chem 271:106539. 10.1016/j.bpc.2020.106539 33508580

[CR78] Sasaki YF, Sekihashi K, Izumiyama F et al (2000) The comet assay with multiple mouse organs: comparison of comet assay results and carcinogenicity with 208 chemicals selected from the IARC monographs and U.S. NTP Carcinogenicity Database. Crit Rev Toxicol 30(6):629–799. 10.1080/10408440008951123 11145306

[CR79] Sasaki YF (2018) Detection of in vitro genotoxicity of pro-mutagens using the comet assay under human and rat liver S9 fractions. MOJ Toxicology 4(4) 10.15406/mojt.2018.04.00109

[CR80] Seo JE, Tryndyak V, Wu Q et al (2019) Quantitative comparison of in vitro genotoxicity between metabolically competent HepaRG cells and HepG2 cells using the high-throughput high-content CometChip assay. Arch Toxicol 93(5):1433–1448. 10.1007/s00204-019-02406-9 30788552

[CR81] Seo J-E, Yu JZ, Xu H et al (2023) Genotoxicity assessment of eight nitrosamines using 2D and 3D HepaRG cell models. Arch Toxicol 97(10):2785–2798. 10.1007/s00204-023-03560-x 37486449 PMC12401554

[CR82] Seo J-E, Le Y, Revollo J et al (2024) Evaluating the mutagenicity of N-nitrosodimethylamine in 2D and 3D HepaRG cell cultures using error-corrected next generation sequencing. Arch Toxicol 98(6):1919–1935. 10.1007/s00204-024-03731-4 38584193 PMC11106104

[CR83] Seo J-E, Xu H, Li X et al (2025a) Genotoxicity evaluation of ten nitrosamine drug substance-related impurities using 2D and 3D HepaRG cell models. Regul Toxicol Pharmacol 162:105906. 10.1016/j.yrtph.2025.105906 40639678 PMC12442021

[CR84] Seo JE, He X, Bryant M et al (2025b) Comparative DNA damage induced by eight nitrosamines in primary human and macaque hepatocytes. Chem Biol Interact 416:111538. 10.1016/j.cbi.2025.111538 40319998 PMC12441990

[CR85] Singh NP, McCoy MT, Tice RR et al (1988) A simple technique for quantitation of low levels of DNA damage in individual cells. Exp Cell Res 175(1):184–191. 10.1016/0014-4827(88)90265-0 3345800

[CR86] Slob W (2014) Benchmark dose and the three Rs. Part II. consequences for study design and animal use. Crit Rev Toxicol 44(7):568–580. 10.3109/10408444.2014.925424 25000331

[CR87] Slob W, Setzer RW (2014) Shape and steepness of toxicological dose-response relationships of continuous endpoints. Crit Rev Toxicol 44(3):270–297. 10.3109/10408444.2013.853726 24252121

[CR88] Steinbrecht S, Kammerer S, Küpper J-H (2019) HepG2 cells with recombinant cytochrome P450 enzyme overexpression: their use and limitation as in vitro liver model. J Cell Biotechnol 5(1):55–64. 10.3233/jcb-189013

[CR89] Tennant RE, Ponting DJ, Thresher A (2023) A deep dive into historical Ames study data for N-nitrosamine compounds. Regul Toxicol Pharmacol 143:105460. 10.1016/j.yrtph.2023.105460 37495012

[CR90] Thomas DN, Wills JW, Tracey H et al (2024) Ames test study designs for nitrosamine mutagenicity testing: qualitative and quantitative analysis of key assay parameters. Mutagenesis 39(2):78–95. 10.1093/mutage/gead033 38112628 PMC10928841

[CR91] Thomas DN, Wills JW, Burman M et al (2025a) Resolution of historically discordant Ames test negative/rodent carcinogenicity positive N-nitrosamines using a sensitive, OECD-aligned design. Mutagenesis 40(2):116–125. 10.1093/mutage/geae027 39485309 PMC12022218

[CR92] Thomas R, Ponting DJ, Thresher A et al (2025b) Critical comparison of BMD and TD50 methods for the calculation of acceptable intakes for N-nitroso compounds. Arch Toxicol 99(3):983–993. 10.1007/s00204-024-03951-8 39751876 PMC11821704

[CR93] U.S. Food & Drug Administration (2023) CDER Nitrosamine Impurity Acceptable Intake Limits. In. https://www.fda.gov/regulatory-information/search-fda-guidance-documents/cder-nitrosamine-impurity-acceptable-intake-limits Accessed 01.09.2025 2025

[CR94] Uhl M, Helma C, Knasmuller S (2000) Evaluation of the single cell gel electrophoresis assay with human hepatoma (Hep G2) cells. Mutat Res 468(2):213–225. 10.1016/s1383-5718(00)00051-6 10882898

[CR95] Vogel M, Carlsson MJ, Skowron C et al (2026) Nitrosamine drug substance-related impurities cause DNA methylation adducts in vitro and in primary hepatocytes upon Cytochrome P450-dependend metabolic activation. Regul Toxicol Pharmacol. 10.1016/j.yrtph.2026.106036 41571032

[CR96] Waldherr M, Misik M, Ferk F et al (2018) Use of HuH6 and other human-derived hepatoma lines for the detection of genotoxins: a new hope for laboratory animals? Arch Toxicol 92(2):921–934. 10.1007/s00204-017-2109-4 29218508 PMC5818615

[CR97] Waskom M (2021) seaborn: statistical data visualization Journal of Open Source Software. vol 6, 0.11.2 edn

[CR98] White PA, Long AS, Johnson GE (2020) Quantitative interpretation of genetic toxicity dose-response data for risk assessment and regulatory decision-making: current status and emerging priorities. Environ Mol Mutagen 61(1):66–83. 10.1002/em.22351 31794061

[CR99] White PA, Zeller A, Pfuhler S, et al. (2019) Re: Gi et al. 2018, In vivo positive mutagenicity of 1,4-dioxane and quantitative analysis of its mutagenicity and carcinogenicity in rats, Archives of Toxicology 92:3207–3221. Archives of toxicology 93(1):211–212 10.1007/s00204-018-2370-1 30539213

[CR100] Wickham H (2016) Ggplot2: elegant graphics for data analysis, 2 edn. Springer International Publishing, Cham, Switzerland

[CR101] Wilkening S, Stahl F, Bader A (2003) Comparison of primary human hepatocytes and hepatoma cell line Hepg2 with regard to their biotransformation properties. Drug Metab Dispos 31(8):1035–1042. 10.1124/dmd.31.8.1035 12867492

[CR102] Wills JW, Johnson GE, Doak SH et al (2016a) Empirical analysis of BMD metrics in genetic toxicology part I: in vitro analyses to provide robust potency rankings and support MOA determinations. Mutagenesis 31(3):255–263. 10.1093/mutage/gev085 26687511

[CR103] Wills JW, Long AS, Johnson GE et al (2016b) Empirical analysis of BMD metrics in genetic toxicology part II: in vivo potency comparisons to promote reductions in the use of experimental animals for genetic toxicity assessment. Mutagenesis 31(3):265–275. 10.1093/mutage/gew009 26984301

[CR104] Winter HK, Ehrlich VA, Grusch M et al (2008) Use of four new human-derived liver-cell lines for the detection of genotoxic compounds in the single-cell gel electrophoresis (SCGE) assay. Mutat Res 657(2):133–139. 10.1016/j.mrgentox.2008.08.012 18790080

[CR105] Yamazaki H, Inui Y, Yun CH et al (1992) Cytochrome P450 2E1 and 2A6 enzymes as major catalysts for metabolic activation of N-nitrosodialkylamines and tobacco-related nitrosamines in human liver microsomes. Carcinogenesis 13(10):1789–1794. 10.1093/carcin/13.10.1789 1423839

[CR106] Zeller A, Pfuhler S, Albertini S et al (2018) A critical appraisal of the sensitivity of in vivo genotoxicity assays in detecting human carcinogens. Mutagenesis 33(2):179–193. 10.1093/mutage/gey005 29669112

[CR107] Zielenska M, Guttenplan JB (1988) Mutagenic activity and specificity of N-nitrosomethylaniline and N-nitrosodiphenylamine in *Salmonella*. Mutat Res 202(1):269–276. 10.1016/0027-5107(88)90189-3 3054528

[CR108] International Council for Harmonisation. (2011). ICH harmonised guideline: S2(R1) Genotoxicity testing and data interpretation for pharmaceuticals intended for human use (Current Step 4 version). https://database.ich.org/sites/default/files/S2%28R1%29%20Guideline.pdf

