# Supplementary material for: The in vitro alkaline comet assay with liver models as a complementary tool for genotoxicity assessment of N-nitrosamines
Source: Arch Toxicol. 2026 Jun 2;100(10):4717–36. doi: 10.1007/s00204-026-04457-1 (PMC13593692; doi:10.1007/s00204-026-04457-1)
Supplement: Supplementary file 1 [file 204_2026_4457_MOESM1_ESM.docx]

# Supplement

## Data gap analysis

Table S1: Number of in vitro studies per N-nitrosamine ordered according to their association to the NA groups in the Mutamind database. The most data rich compounds are marked in bold. Only N-nitrosamines according to the CPCA definition are shown.

| **Name** | **CAS** | **Studies [n]** | **CPCA** |
| --- | --- | --- | --- |
| *N*-Nitrosodipropylamine | 621-64-7 | 2 | 1 |
| *N*-Nitrosodiethylamine | 55-18-5 | **22** | 1 |
| *N*-Nitrosodimethylamine | 62-75-9 | **55** | 1 |
| *N*-Nitrosoethylmethylamine | 10595-95-6 | 3 | 1 |
| 4-(Methylnitrosamino)-1-(3-pyridyl)-1-(butanone) NNK | 64091-91-4 | 9 | 1 |
| Methyl(acetoxymethyl)nitrosamine | 56856-83-8 | 6 | 1 |
| [(Acetoxymethyl)nitrosamino]-1-(3-pyridyl)-1-butanone | 127686-49-1 | **13** | 1 |
| *N*-Nitroso-di-n-butylamine | 924-16-3 | 5 | 1 |
| *N*-Nitrosomorpholine | 59-89-2 | **11** | 2 |
| 1-Nitroso-4-methylpiperazine | 16339-07-4 | 2 | 3 |
| 3-*N*-nitroso-oxazolidin-2-one | 39884-52-1 | 2 | 3 |
| *N*-Nitrosopiperidine | 100-75-4 | **10** | 3 |
| *N*-Nitrosodiethanolamine | 1116-54-7 | 1 | 3 |
| *N*-Nitrosonornicotine | 80508-23-2 | 4 | 4 |
| *N*-Nitrosopyrrolidine | 930-55-2 | **10** | 4 |
| *N*-Nitrosodiphenylamine | 86-30-6 | 1 | 5 |
| *N*-Nitrosodicyclohexylamine | 947-92-2 | 1 | 5 |

## Metabolic activation

Table S2: Final calculated metabolic ratios of the rat and human cell systems. For nicotine no metabolic ratio could be determined, as there was no signal for nicotine but for cotinine. Here, total areas of cotinine are listed as surrogate parameter.

| **Enzyme** | **Substrate** | **Metabolic conv. PRHs [%]** | **Metabolic conv. PHHs [%]** | **Metabolic conv. HepG2 [%]** |
| --- | --- | --- | --- | --- |
| CYP2B6 | *Bupropione* | 0.60 | 0.73 | not detected |
| CYP2E1 | *Chlorzoxazone* | 0.20 | not detected | not detected |
| CYP2D6 | *Dextromethorphan* | 1.83 | 1.01 | not detected |
| CYP2C19 | *Mephenytoin* | 0.01 | not detected | not detected |
| CYP3A4 | *Midazolam* | 0.09 | 0.07 | not detected |
| CYP2A6 | *Nicotine* | (96.41) | 12.73 | not detected |
| CYP1A2 | *Phenacetine* | 0.26 | 0.25 | not detected |
| CYP2C9 | *Tolbutamide* | 1.75 | 0.64 | not detected |

Table S3: Final metabolic ratios of rat and hamster S9-mixtures.

| **Enzyme** | **Substrate** | **Metabolic conversion  rat S9-mix [%]** | **Metabolic conversion hamster S9-mix [%]** |
| --- | --- | --- | --- |
| CYP2B6 | *Bupropione* | 98.30 | 91.40 |
| CYP2E1 | *Chlorzoxazone* | 19.40 | 15.10 |
| CYP2D6 | *Dextromethorphan* | 50.30 | 83.30 |
| CYP2C19 | *Mephenytoin* | 0.06 | 0.40 |
| CYP3A4 | *Midazolam* | 24.20 | 6.00 |
| CYP2A6 | *Nicotine* | 53.50 | 94.00 |
| CYP1A2 | *Phenacetine* | 7.00 | 7.40 |
| CYP2C9 | *Tolbutamide* | 4.00 | 2.80 |

Table S4: Final ion transitions, which were integrated into the multiple reaction monitoring (MRM) experiment for substrate and compound semi quantification.

|  | **ID** | **Q1** | **Q3** | **dwell time [msec]** | **Declustering potential [eV]** | **Collision energy [eV]** |
| --- | --- | --- | --- | --- | --- | --- |
| (+)MRM | Tolbutamide | 271.1 | 155.1 | 5 | 80 | 27 |
|  | Hydroxytolbutamide-1 | 287.1 | 171 | 55 | 80 | 23 |
|  | Hydroxytolbutamide-2 | 287.1 | 188 | 55 | 80 | 23 |
|  | Phenacetin | 180 | 110 | 5 | 80 | 25 |
|  | Acetaminophen-1 | 152 | 110 | 55 | 80 | 25 |
|  | Acetaminophen-2 | 152 | 93 | 55 | 80 | 25 |
|  | Mephenytoin | 219.4 | 134.2 | 5 | 80 | 28 |
|  | Hydroxymephenytoin-1 | 235 | 150 | 55 | 80 | 28 |
|  | Hydroxymephenytoin-2 | 235 | 133 | 55 | 80 | 28 |
|  | Dextromethorphan | 272.2 | 171.1 | 5 | 80 | 40 |
|  | Dextrorphan-1 | 258.18 | 199.2 | 55 | 80 | 40 |
|  | Dextrorphan-2 | 258.18 | 157.1 | 55 | 80 | 40 |
|  | Bupropion | 240.1 | 184 | 5 | 80 | 22 |
|  | Hydroxybupropion-1 | 256.2 | 167 | 55 | 80 | 35 |
|  | Hydroxybupropion-2 | 256.2 | 131 | 55 | 80 | 35 |
|  | Midazolam | 326.2 | 291.1 | 5 | 80 | 35 |
|  | Hydroxymidazolam-1 | 343 | 297.1 | 55 | 80 | 30 |
|  | Hydroxymidazolam-2 | 343 | 203.2 | 55 | 80 | 30 |
|  | Nicotine | 163 | 130.1 | 5 | 80 | 25 |
|  | Cotinine-1 | 177.1 | 98 | 55 | 80 | 30 |
|  | Cotinine-2 | 177.1 | 80 | 55 | 80 | 30 |
| (-)MRM | Chlorzoxazone | 168.1 | 132.1 | 5 | -100 | -50 |
|  | 6-Hydroxychlorzoxazone-1 | 184.1 | 120 | 55 | -100 | -40 |
|  | 6-Hydroxychlorzoxazone-2 | 184.1 | 139.1 | 55 | -100 | -40 |
|  |  |  |  |  |  |  |
|  |  | Quantifier |  |  |  |  |
|  |  | Qualifier |  |  |  |  |

Figure S1: mRNA expression of CYP enzymes in primary human hepatocytes (PHH) as compared to human liver tissue. Data are represented as mean ± SEM (n = 4-5).

## Comet assay HepG2 cells

Figure S2: Distribution of N-nitroso desloratadine (NND, 1246819-22-6) log_10_ median data tested in HepG2 with hamster S9-mix and the following concentration range: 0, 0.005, 0.01, 0.02, 0.05, 0.1, 0.25 mM. The smaller circles represent the median per replicate for each concentration and the diamond their mean. The x-axis denotes the log_10_ concentration [mM] and the y-axis the log10 the tail intensity [%]. The horizontal line represents the fold-change from which a positive result exists (2-fold). All means above the line are also statistically significant different compared to the control with p > 0.05. Precipitation occurs in the four highest concentrations (0.05, 0.1, 0.2, 0.25 mM). To represent the control on the logarithmic scale, a value of -2.5 was specified.

Figure S3: Distribution of N-nitrosodiethanolamine (NDELA, 1116-54-7) log_10_ median data tested in HepG2 with rat S9-mix and the following concentration range: mM. The smaller circles represent the median per replicate for each concentration and the diamond their mean. The x-axis denotes the log10 concentration [mM] and the y-axis the log10 the tail intensity [%]. The horizontal line represents the fold-change from which a positive result exists (2-fold). All means above the line are also statistically significant different compared to the control with p > 0.05. To represent the control on the logarithmic scale, a value of -0.6 was specified.

Figure S4: Distribution of Methyl-t-butylnitrosamine (NMtBu, 2504-18-9) log_10_ median data tested in HepG2 with hamster S9-mix and the following concentration range: 0, 0.5, 1, 2, 5, 10 mM. The smaller circles represent the median per replicate for each concentration and the diamond their mean. The x-axis denotes the log10 concentration [mM] and the y-axis the log10 the tail intensity [%]. The horizontal line represents the fold-change from which a positive result exists (2-fold). All means above the line are also statistically significant different compared to the control with p > 0.05. To represent the control on the logarithmic scale, a value of -1 was specified.

## BMD modeling

Figure S5: BMD modeling of PHH in vitro comet assay data: **(A)** The model with the highest weight (Expon. m5-abv) is shown per NA before model averaging using PROAST 70.3 with a covariate approach. The “critical effect size” of 100 is shown as a dotted line. The x-axis denotes the log_10_ concentration [mM] and the y-axis the log_10_ the tail intensity [%]. N-Nitrosodimethylamine (NDMA, all62759), N-Nitrosofluoxetine (NFluo, 150494067), N-Nitrosodiethanolamine (NDELA, 1116547) and 2-Propanamine, N-methyl-N-nitroso- (NMIPA, 30533085). The smaller triangles represent the replicates for each concentration and the big triangles their mean. Expon. = exponential; Inv. Expon. = inverse exponential; LN = lognormal DR. **(B)** quantile-quantile plot shown representatively on log_10_ scale. **(C)** Weights used in model averaging per model.

Figure S6: BMD modeling of PRH in vitro comet assay data: **(A)** The model with the highest weight (Expon. m5-abv) is shown per NA before model averaging using PROAST 70.3 with a covariate approach. The “critical effect size” of 100 is shown as a dotted line. The x-axis denotes the log_10_ concentration [mM] and the y-axis the log_10_ the tail intensity [%]. N-Nitrosodimethylamine (NDMA, all62759), N-Nitrosofluoxetine (NFluo, 150494067), N-Nitrosodiethanolamine (NDELA, 1116547) and 2-Propanamine, N-methyl-N-nitroso- (NMIPA, 30533085). The smaller triangles represent the replicates for each concentration and the big triangles their mean. Expon. = exponential; Inv. Expon. = inverse exponential; LN = lognormal DR. **(B)** quantile-quantile plot shown representatively on log_10_ scale. **(C)** Weights used in model averaging per model.

Figure S5: BMD modeling of HepG2 with hamster S9-mix in vitro comet assay data: **(A)** The model with the highest weight (Inv. Expon. m5-abv) is shown per NA before model averaging using PROAST 70.3 with a covariate approach. The “critical effect size” of 100 is shown as a dotted line. The x-axis denotes the log_10_ concentration [mM] and the y-axis the log_10_ the tail intensity [%]. N-Nitrosodimethylamine (NDMA, all62759), N-Nitrosofluoxetine (NFluo, 150494067), N-Nitrosodiethanolamine (NDELA, 1116547), N-Nitrosonornicotine (2S) (S-NNN, 16543558) and 2-Propanamine, N-methyl-N-nitroso- (NMIPA, 30533085). The smaller triangles represent the replicates for each concentration and the big triangles their mean. Expon. = exponential; Inv. Expon. = inverse exponential; LN = lognormal DR. **(B)** quantile-quantile plot shown representatively on log_10_ scale. **(C)** Weights used in model averaging per model.

Figure S6: BMD modeling of HepG2 with rat S9-mix in vitro comet assay data: **(A)** The model with the highest weight (Expon. 5-abv) is shown per NA before model averaging using PROAST 70.3 with a covariate approach. The “critical effect size” of 100 is shown as a dotted line. The x-axis denotes the log_10_ concentration [mM] and the y-axis the log_10_ the tail intensity [%]. N-Nitrosodimethylamine (NDMA, all62759), N-Nitrosofluoxetine (NFluo, 150494067) and methyl-t-butylnitrosamine (NMtBu, 2504189). The smaller triangles represent the replicates for each concentration and the big triangles their mean. Expon. = exponential; Inv. Expon. = inverse exponential; LN = lognormal DR. **(B)** quantile-quantile plot shown representatively on log_10_ scale. **(C)** Weights used in model averaging per model.
